# The gut microbiome is a significant risk factor for future chronic lung disease

**DOI:** 10.1016/j.jaci.2022.12.810

**Published:** 2023-04

**Authors:** Yang Liu, Shu Mei Teo, Guillaume Méric, Howard H.F. Tang, Qiyun Zhu, Jon G. Sanders, Yoshiki Vázquez-Baeza, Karin Verspoor, Ville A. Vartiainen, Pekka Jousilahti, Leo Lahti, Teemu Niiranen, Aki S. Havulinna, Rob Knight, Veikko Salomaa, Michael Inouye

**Affiliations:** a Department of Clinical Pathology, Melbourne Medical School, The University of Melbourne, Melbourne, Australia; b Cambridge Baker Systems Genomics Initiative, Baker Heart and Diabetes Institute, Melbourne, Australia; c Cambridge Baker Systems Genomics Initiative, Department of Public Health and Primary Care, University of Cambridge, Cambridge, United Kingdom; d Centre for Youth Mental Health, University of Melbourne, Melbourne, Australia; e School of Life Sciences, Arizona State University, Tempe, Ariz; f Biodesign Center for Fundamental and Applied Microbiomics, Arizona State University, Tempe, Ariz; g Department of Ecology and Evolutionary Biology, Cornell University, Ithaca, NY; h Center for Microbiome Innovation, Jacobs School of Engineering, University of California San Diego, La Jolla, Calif; i School of Computing Technologies, RMIT University, Melbourne, Australia; j School of Computing and Information Systems, The University of Melbourne, Melbourne, Australia; k Department of Public Health and Welfare, Finnish Institute for Health and Welfare, Helsinki, Finland; l Individualized Drug Therapy Research Program, Faculty of Medicine, University of Helsinki, Helsinki, Finland; m Department of Pulmonary Medicine, Heart and Lung Center, Helsinki University Hospital, Helsinki, Finland; n Department of Computing, University of Turku, Turku, Finland; o Division of Medicine, Turku University Hospital and University of Turku, Turku, Finland; p Institute for Molecular Medicine Finland, FIMM-HiLIFE, University of Helsinki, Helsinki, Finland; q Department of Computer Science and Engineering, University of California San Diego, La Jolla, Calif; r Department of Pediatrics, School of Medicine, University of California San Diego, La Jolla, Calif; s British Heart Foundation Cardiovascular Epidemiology Unit, Department of Public Health and Primary Care, University of Cambridge, Cambridge, United Kingdom; t British Heart Foundation Cambridge Centre of Research Excellence, School of Clinical Medicine, University of Cambridge, Cambridge, United Kingdom; u Health Data Research UK Cambridge, Wellcome Genome Campus and University of Cambridge, Cambridge, United Kingdom; v The Alan Turing Institute, London, United Kingdom; w Heart and Lung Research Institute, University of Cambridge, Cambridge, United Kingdom

**Keywords:** Gut, microbiome, metagenomics, asthma, COPD

## Abstract

**Background:**

The gut-lung axis is generally recognized, but there are few large studies of the gut microbiome and incident respiratory disease in adults.

**Objective:**

We sought to investigate the association and predictive capacity of the gut microbiome for incident asthma and chronic obstructive pulmonary disease (COPD).

**Methods:**

Shallow metagenomic sequencing was performed for stool samples from a prospective, population-based cohort (FINRISK02; N = 7115 adults) with linked national administrative health register–derived classifications for incident asthma and COPD up to 15 years after baseline. Generalized linear models and Cox regressions were used to assess associations of microbial taxa and diversity with disease occurrence. Predictive models were constructed using machine learning with extreme gradient boosting. Models considered taxa abundances individually and in combination with other risk factors, including sex, age, body mass index, and smoking status.

**Results:**

A total of 695 and 392 statistically significant associations were found between baseline taxonomic groups and incident asthma and COPD, respectively. Gradient boosting decision trees of baseline gut microbiome abundance predicted incident asthma and COPD in the validation data sets with mean area under the curves of 0.608 and 0.780, respectively. Cox analysis showed that the baseline gut microbiome achieved higher predictive performance than individual conventional risk factors, with C-indices of 0.623 for asthma and 0.817 for COPD. The integration of the gut microbiome and conventional risk factors further improved prediction capacities.

**Conclusions:**

The gut microbiome is a significant risk factor for incident asthma and incident COPD and is largely independent of conventional risk factors.

Asthma and chronic obstructive pulmonary disease (COPD) represent the vast majority of chronic respiratory diseases worldwide, causing a considerable burden on health and economy.^1^^,^^2^ Both asthma and COPD are recognized as heterogeneous diseases with diverse phenotypes and various underlying mechanisms.^3^, ^4^, ^5^, ^6^ Currently, spirometry-confirmed airflow limitation is the most common reference standard for establishing diagnoses of asthma and COPD, yet a negative spirometry test result does not rule out the disease.^7^^,^^8^ Other criteria that complement evaluation include self-reported symptoms, medical history, physical examination, and other diagnoses such as infection, interstitial lung disease, and others.^7^^,^^9^ Despite rapidly changing assessments and treatments, both asthma and COPD remain largely underdiagnosed and thus undertreated, leading to lesser quality of life and poorer disease outcomes.^3^^,^^9^

With recent advances in high-throughput sequencing, improved characterization of the human respiratory and gastrointestinal microbiome has been followed by growing recognition of the link between human microbiota and chronic respiratory disease.^10^^,^^11^ The gut microbiome is by far the largest and most studied microbial community in the human body.^11^^,^^12^ Although the lung microbiome has become well characterized only recently, the link between the lung microbiome and respiratory diseases has been generally acknowledged.^10^^,^^13^, ^14^, ^15^ “Dysbiotic” changes in both airway and gut microbiome have been linked to respiratory diseases; however, the precise mechanism or causal pathway is, as yet, not well understood.^16^, ^17^, ^18^, ^19^ Emerging evidence suggests cross-talk between gut microbiome and the lungs, via changes to immune responses as well as an interaction of microbiota between the sites, in a hypothesized “gut-lung axis.”^11^^,^^20^

Existing studies on the association between gut microbiota and asthma have focused mainly on disease development during childhood,^21^, ^22^, ^23^ which is driven by evidence of the influence of early-life microbial exposures on immune function.^24^^,^^25^ Previous cross-sectional studies have reported compositional and functional differences of the gut microbiome between adult patients with asthma and healthy controls.^26^, ^27^, ^28^, ^29^ However, little is known about whether and to what extent the gut microbiome affects the prospective risk of developing incident asthma in adults. For COPD, there have been far fewer studies on the link between the gut microbiome and disease. Recently, the first analysis of gut microbiome in COPD by Bowerman et al^30^ reported that the fecal microbiome and metabolome differentiate patients with COPD and healthy controls, which suggests a possible avenue for further investigation using prospective population-scale data sets. Finally, it is only in recent years that methodological and technological advances have opened up the possibility of using large-scale microbial data to predict human respiratory disease,^22^^,^^31^ but the feasibility of such measures is yet to be evaluated for COPD.

Here, we report association analysis and predictive modeling of the gut microbiome and incident asthma and COPD using stool samples from more than 7000 participants of a prospective population-based cohort (FINRISK 2002) with electronic health records (EHRs) over approximately 15 years of follow-up.^32^ Specifically, we (1) describe the gut microbial composition from shallow shotgun metagenomic sequencing and assess the associations with incident asthma and COPD, (2) use machine learning approaches to quantify the predictive capacities of the gut microbiome at baseline for incident respiratory disease, and (3) construct integrated models of the gut microbiome and conventional risk factors and evaluate their predictive performance.

## Methods

### Study design and participants

The FINRISK 2002 study was a population-based nationwide survey carried out in Finland in 2002, consisting of random samples of the population aged 25 to 74 years drawn from the National Population Information System.^32^ The sampling was stratified by sex, region, and 10-year age group so that each stratum had 250 participants. The survey included self-administered questionnaires, health examinations conducted at the study sites by trained personnel, and collection of biological samples. The overall participation rate was 65.5% (n = 8798). The participants were followed up through linkage to national administrative electronic registers that proved highly reliable.^33^, ^34^, ^35^ Inclusion criteria have been described elsewhere.^32^ The present study excluded participants who did not have linked EHRs of clinical end points, who had prior diagnoses of the diseases for prediction, who were pregnant at baseline, and who had prescription of antibiotics use defined as ATC code of J01 up to 6 months before baseline. The incident cases of asthma and COPD were identified according to *International Classification of Diseases, Tenth Revision* diagnosis codes (Finnish modification) from linked EHRs, which were last followed up by December 31, 2016. COPD cases were defined using *International Classification of Diseases* codes J43|J44; asthma cases were defined using *International Classification of Diseases* codes J45|J46, or the Social Insurance Institution of Finland (Kela) reimbursement code 203 for asthma medication, or medicine purchases with *Anatomical Therapeutic Chemical* codes R03BA|R03BC|R03DC|R03AK. Covariates included baseline age, sex, body mass index (BMI), and smoking. Written informed consent was obtained from all participants. The Coordinating Ethics Committee of the Helsinki and Uusimaa Hospital District approved the FINRISK 2002 study protocols (reference no. 558/E3/2001). The study was conducted according to the World Medical Association’s Declaration of Helsinki on ethical principles.

### Sample collection

During the baseline survey, stool samples were collected by willing participants at home using an ad hoc kit constructed in-house in the Finnish Institute for Health and Welfare (THL) with detailed instructions and a scoop method. The participants were advised to collect the sample preferably in the morning, but any time convenient to the participant was considered acceptable. The samples were mailed overnight between Monday and Thursday to the laboratory of the Finnish Institute for Health and Welfare for storing at −20°C. Special care was taken to avoid delayed transit at the post office over the weekend. The sample collection was done under winter conditions, with average temperatures well below 0°C in Finland from January through March 2002, and no special arrangements were made regarding the temperature during transportation. The possible short-term exposure of samples to room temperature after collection and the extended time before freezing might cause variations in detection and relative abundances of rare taxa,^36^^,^^37^ which is relatively minor given the average environmental temperatures well below 0°C and the focus on common taxa in this study. The frozen stool samples were transferred to University of California San Diego for sequencing in 2017.

### DNA extraction, sequence processing, and taxonomic profiling

DNA extraction was carried out using the MagAttract PowerSoil DNA kit (Qiagen, Venlo, The Netherlands) according to the Earth Microbiome Project protocols.^38^ Libraries were prepared using a miniaturized version of the KAPA HyperPlus Illumina-compatible prep kit according to manufacturer’s protocol.^39^ The extracted DNA was normalized to 5 ng total input per sample with an Echo 550 acoustic liquid-handling robot (Labcyte Inc, San Jose, Calif). A 1/10 scale enzymatic fragmentation, end-repair, and adapter-ligation reactions were performed with a Mosquito HV liquid-handling robot (TTP Labtech, Melbourn, United Kingdom). Sequencing adapters were performed on the basis of the iTru protocol^40^ by ligating universal adapter stubs and adding sample-specific barcoded sequences in a subsequent PCR step. Amplified and barcoded libraries were quantified with the PicoGreen assay and pooled in approximately equimolar ratios. An Illumina HiSeq 4000 instrument was used to perform shallow shotgun metagenomics sequencing^41^ to a mean depth of approximately 10^6^ reads/sample. The stool shotgun sequencing was successfully performed in 7231 individuals. The metagenomic sequences were processed using an automated Snakemake workflow pipeline (https://github.com/tanaes/snakemake_assemble).^39^^,^^42^ Removal of low-quality sequences and adapters was performed using Atropos.^43^ Host reads were removed with Bowtie2^44^ by mapping against human genome assembly GRCh38. Samples with total reads lower than 400,000 were removed to preserve the quality of data while retaining most of the disease cases.

The raw shotgun metagenomes were mapped to an index database based on taxonomic nomenclature introduced and updated in the Genome Taxonomy Database (GTDB) release 89^45^ using default parameters in the k-mer–based metagenomic classification tool Centrifuge 1.0.4.^46^ In total, 151 phyla, 338 classes, 925 orders, 2,254 families, 7,906 genera, and 24,705 species were uniquely identified on the basis of GTDB taxonomy. The relative abundances of a bacterial taxon at phylum, class, order, family, genus, and species levels were computed as the proportion of reads assigned to the clade rooted at this taxon among total classified reads. The relative abundance of a taxon with no reads assigned was considered as zero in the metagenome. The present analyses focused on common and relatively abundant microbial taxa with relative abundances greater than 0.01% in more than 1% of samples. Three measures of microbial diversity were calculated: Shannon’s alpha diversity, Chao1 richness, and Pielou’s evenness (R packages vegan v2.5.5 and otuSummary v0.1.1). To overcome the sample comparison biases of compositional data, the centered log-ratio (CLR) transformation was performed on abundance data (R package compositions v1.4.2), of which zeros were substituted with 1/10 of nonzero minimum abundance.

### Machine learning and statistical analysis

A machine learning framework was used to develop prediction models at different taxonomic levels separately. The samples were randomly partitioned into 2 subsets: (1) a training data set (70% of samples) for developing models and (2) a validation data set (30% of samples) for evaluating prediction performance. We resampled the data 10 times and performed the same training and validation procedure for each sampling partition. In each training data set, we first selected microbial indicators for predicting incident asthma and COPD; we analyzed the relationships between taxon-level abundance and incident disease using logistic regression adjusted for age and sex, Cox regression for time to disease onset adjusted for age and sex, and Spearman correlation. These approaches have been widely used to explore the relationship between microbiome features and disease-related traits in previous studies.^30^^,^^31^^,^^47^ Logistic regression naturally models binary outcomes; Cox regression takes into account the time until events occur; Spearman correlation is a nonparametric measure of the strength and direction of nonlinear correlation. To include any taxa with potential predictive signals, we considered taxa that were associated with incident diseases at a significance threshold of *P* less than .05 by any of the above approaches for further analyses. To avoid overfitting of feature selection, we did not use algorithms that take into account all the features simultaneously. The selected taxa together with diversity measurements were considered as microbial predictors for developing gradient boosting decision tree model, an ensemble method of sequential and additive training of trees. Each tree fits the residuals of the previous tree in sequence to minimize errors, which makes gradient boosting a highly efficient method. In addition, gradient boosting decision trees are robust to correlated features that naturally exist in microbiome abundance data and apply regularization to reduce overfitting. Gradient boosting decision tree models were implemented with XGBoost 0.82 through 5-fold cross-validation to determine optimal hyperparameters with Bayesian optimization (R package mlrMBO 1.1.2). XGBoost models were developed with objective “binary:logistic” and parameters considered include eta[0.001, 0.5], max_depth[3,10], min_child_weight[5,100], subsample[0.8,0.95], colsample_bytree[0.6,0.85], gamma[0,5], lambda[0.0001, 1], max_delta_step[0,8], early_stopping_rounds[10,20], and nrounds[100,1500]. The optimal setting was then trained on the whole training data to build the final model used in validation. For asthma and COPD, the predicted values from the optimal gradient boosting model of gut microbial features were used as the gut microbiome scores in the validation data set where the scores were used for further Cox analyses for each disease condition. We additionally performed ridge logistic regression to compare the prediction performance using the same samples for training and testing. The gradient-boosted trees-based models outperformed those based on ridge logistic regression. A similar trend of prediction performance across taxonomic levels was observed with both methods. The final performance across various models and partitions was assessed in the validation data sets.

Wilcoxon rank-sum test was performed to compare differences in patient characteristics and microbial diversity metrics between incident cases and noncases across all samples for each disease. Cox regression with adjustment of age and sex was used to assess the association between taxon-level CLR abundance and incident disease using all samples. Benjamini-Hochberg correction was used to control for multiple testing at each taxonomic rank, and false-discovery rate less than 0.05 was considered as statistical significance. We additionally applied Benjamini-Yekutieli correction across all taxonomic levels and reported corrected *P* values.

Cox models of conventional risk factors and in combination with the gut microbiome score were built using the time from baseline to the occurrence of the disease or end of follow-up in the validation data set (R package survival 2.44). Sensitivity analyses considered income and education level as risk factors and were performed in the validation data set using samples with complete data of risk factors. Association of risk factors was assessed separately and in combination using Cox models for incident asthma and COPD. Education was classified into 3 groups, low, middle, and high education, on the basis of years at school tertiles adjusted for birth cohort as reported in the questionnaire. Income level was represented as an ordinal variable of 1 to 9 according to household’s income before tax deduction with the following cutoffs: less than 50,000 FIM (<8,400€), 50,001 to 100,000 FIM (8,401-16,820€), 100,001 to 150,000 FIM (16,821-25,230€), 150,001 to 200,000 FIM (25,231-33,640€), 200,001 to 250,000 FIM (33,641-42,050€), 250,001-300,000 FIM (42,051-50,460€), 300,001-350,000 FIM (50,461-58,870€), 350,001-400,000 FIM (58,871-67,280€), and more than 400,000 FIM (>67,280€). Statistical analyses were carried out with R 3.6.1.

### Data and code availability

The FINRISK data for the present study are available with a written application to the THL Biobank as instructed on the website of the Biobank (https://thl.fi/en/web/thl-biobank/for-researchers). A separate permission is needed from FINDATA (https://www.findata.fi/en/) for use of the EHR data. Custom code for analysis in this study is available at https://github.com/dpredprj/gut_respiratory_link.

## Results

A total of 7115 FINRISK02 participants with baseline gut microbiome profiles and EHR linkage were available for the present study. A summary description of the cohort is given in the Methods section, and baseline characteristics are reported in Table I. After quality control and exclusion criteria were applied, 435 and 145 incident cases of asthma and COPD, respectively, occurred during a median follow-up of 14.8 years after gut microbiome sampling at baseline. Notably, more males than females developed COPD, and incident COPD cases displayed older baseline age than noncases (*P* < .001). The age of onset of incident COPD was older compared with that of incident asthma (*P* < .001). A higher BMI was observed in asthma cases versus noncases (*P* = .002), whereas there was no difference in BMI between COPD cases and noncases. For both COPD and asthma, a higher proportion of current smokers during the survey year were observed in disease cases than in noncases.

**Table I:** Characteristics of study participants

Characteristic	Asthma		COPD	
	Incident cases (n = 435)	Noncases (n = 5244)	Incident cases (n = 145)	Noncases (n = 5932)
Sex: female, n (%)	252 (57.9)	2740 (52.3)	43 (29.7)	3204 (54)
Baseline age (y)	50.9 (40.5-60.5)	50.5 (39.2-59.3)	59.5 (53.6-66.5)	50.5 (39.2-59.5)
Age at first event (y)	57.6 (46.7-67.3)	—	69.1 (61.3-73.6)	—
BMI (kg/m^2^)	26.7 (24-30.7)	26.3 (23.7-29.3)	26.6 (23.5-29.6)	26.4 (23.7-29.5)
Current smoker, n (%)	151 (34.8)	1192 (22.8)	105 (72.9)	1313 (22.2)
Ex-smoker, n (%)	94 (21.6)	1181 (22.5)	32 (22.1)	1321 (22.3)

Continuous variables are presented as median (interquartile range).

### Gut microbiome composition and taxon-level abundances

**Fig 1:**
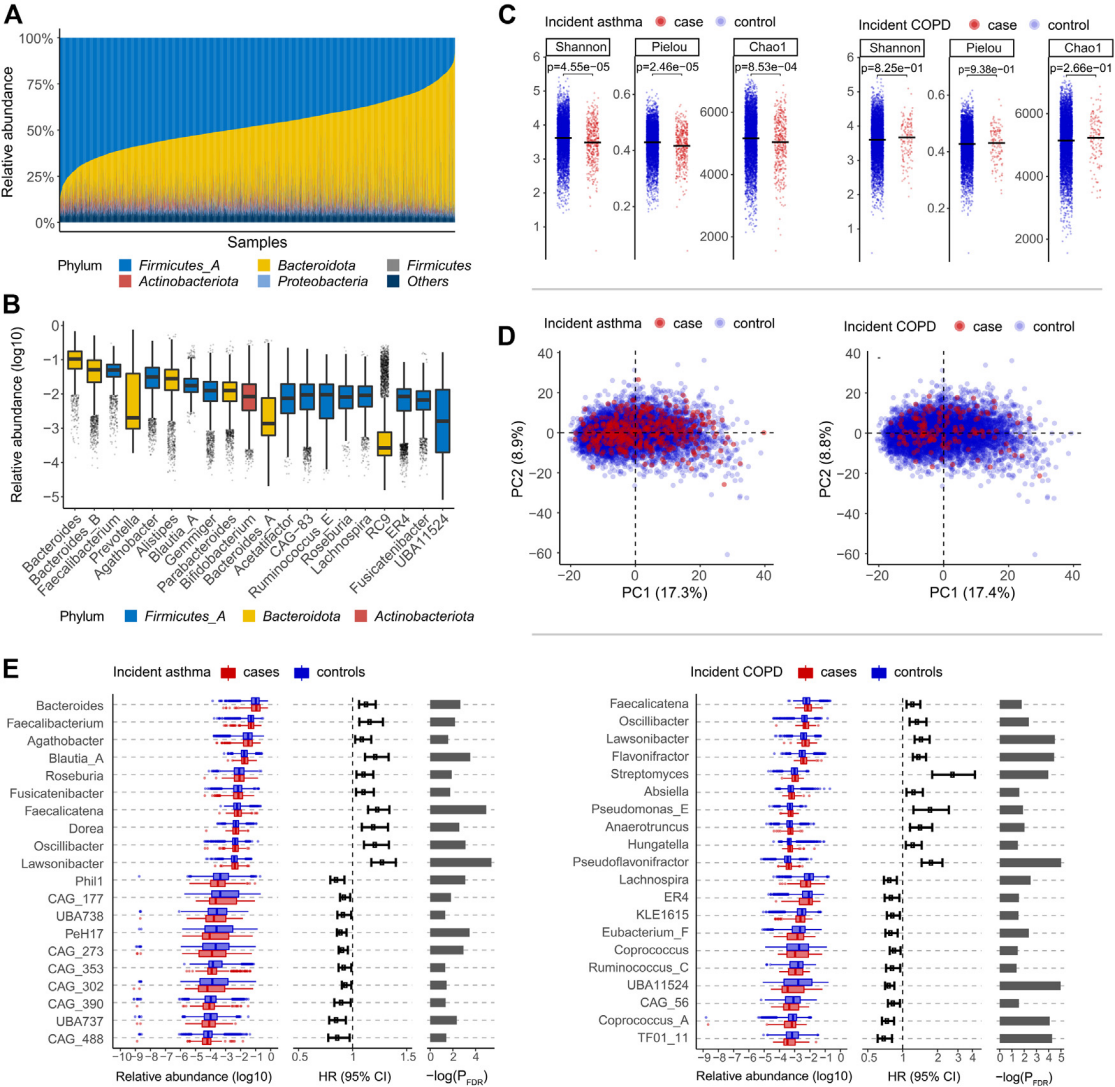
Gut microbiome composition and characteristics. **A,** Gut microbiome profiles at phylum level. **B,** Box plots of the 20 most abundant genera sorted by mean relative abundance. **C,** Shannon’s, Pielous’s, and Chao1 indices at genus level between cases and noncases. Median values are represented by horizontal lines. **D,** Principal-component analysis on CLR-transformed abundances at genus level. **E,** Genera associated with incident asthma or COPD surpassing a false-discovery rate threshold of 5% (PFDR < 0.05) with Benjamini-Hochberg correction. Only the top 10 most abundant genera for each of combination of positive or negative associations, with COPD or asthma.

Individual gut microbiome compositions were characterized by shallow shotgun metagenomic sequencing of stool samples (see the Methods section). The present study focused on microbial taxa whose relative abundance exceeded 0.01% in at least 1% of samples; this yielded 46 phyla, 71 classes, 124 orders, 232 families, 617 genera, and 1224 species, as classified according to the Genome Taxonomy Database (GTDB) release 89.^45^ Most of the gut microbiota were dominated by the Firmicutes_A and Bacteroidota phyla (Fig 1, *A*), which mostly comprised members of classes Clostridia and Bacteroidia, respectively. At the genus level, *Faecalibacterium* and *Agathobacter* in phylum Firmicutes_A, as well as *Bacteroides*, *Bacteroides_B*, and *Prevotella* in phylum Bacteroidota, were most abundant in most samples (Fig 1, *B*).

Baseline alpha-diversity measures differed between incident asthma cases and noncases (*P* < .01), with lower values of Shannon’s, Chao1, and Pielou’s indices in individuals who went on to develop asthma (Fig 1, *C*). There was no statistical difference in alpha-diversity indices between COPD cases and noncases. Principal- component analysis of the CLR-transformed abundances showed no clear separation between incident cases and noncases (Fig 1, *D*), suggesting that the association of incident asthma and COPD with the gut microbiome was unlikely related to the whole microbial community and may be attributable to specific microbial taxa.

We assessed the association between baseline taxon-level microbial abundances and incident respiratory diseases using Cox regression, based on CLRs (see the Methods section). At 5% false-discovery rate, statistically significant associations of incident asthma were found in 5 phyla, 5 classes, 18 orders, 111 families, 257 genera, and 299 species (see Table E1 in this article’s Online Repository at www.jacionline.org); for incident COPD, we found associations with 5 phyla, 7 classes, 32 orders, 57 families, 133 genera, and 158 species (see Table E2 in this article’s Online Repository at www.jacionline.org). Of the asthma- and COPD-associated taxa, 76% and 68.6% showed positive associations with disease incidence, respectively. A number of highly abundant genera were associated with incident asthma, such as *Bacteroides*, *Faecalibacterium*, *Agathobacter*, *Blautia_A*, and *Roseburia* (Fig 1, *E*). Among the most abundant COPD-associated genera, increased abundance of *Faecalicatena*, *Oscillibacter*, *Lawsonibacter*, *Flavonifractor*, and *Streptomyces* and reduced abundances of *Lachnospira*, *ER4*, *KLE1615*, *Eubacterium_F*, and *Coprococcus* were associated with incident COPD.

### Gut microbiome and gradient boosting decision trees to predict incident asthma and COPD

To investigate whether the baseline gut microbiome was predictive of incident asthma and COPD, we train and validate prediction models via the machine learning algorithm of gradient boosting decision trees. These models were trained with 5-fold cross-validation in 70% of the individuals and then the performances were validated in the remaining 30% (see the Methods section); all performance metrics given are based on the 30% validation set unless otherwise specified. Models were developed at different taxonomic levels separately and for a combination of all taxonomic levels (see Fig E1 in this article’s Online Repository at www.jacionline.org). To assess sampling variation, we resampled training and testing partitions at different taxonomic levels 10 times and report mean values of prediction performance.

The best performance was obtained at individual taxonomic levels, rather than their combination, for both asthma and COPD prediction. Generally better prediction performance was attained at lower taxonomic levels, particularly for COPD where the highest average area under the operating characteristic curve (AUC) was at species level (mean AUC, 0.780), followed by genus (mean AUC, 0.734) and family (mean AUC, 0.688) levels. For prediction of incident asthma, the best performance was obtained at family level (mean AUC, 0.608), with slight attenuation of AUC scores obtained at genus (mean AUC, 0.592) and species (mean AUC, 0.593) levels.

### The gut microbiome had greater predictive value than individual conventional risk factors

**Fig 2:**
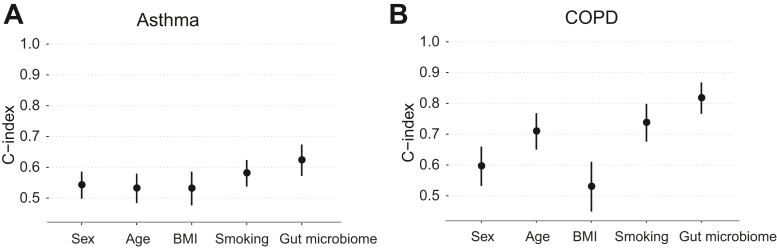
Predictive capacity of each risk factor separately for (**A**) incident asthma or (**B**) COPD. Univariate Cox models were used for each of sex, baseline age, BMI, smoking, and gut microbiome individually. Points and error bars represent the C-indices and 95% CIs.

To compare the predictive value of conventional risk factors and the gut microbiome for incident asthma and COPD, we first conducted univariate analysis using Cox models. We used the optimal cross-validated gradient boosting model at family and species level for asthma and COPD, respectively, and refer to the resultant score as a “gut microbiome score” for each condition. We found that the gut microbiome score had a relatively high predictive capacity with C-indices of 0.623 for asthma and 0.817 for COPD, which were each greater than those of other risk factors (Fig 2). Smoking status at baseline was associated with increased risk of both asthma (hazard ratio [HR], 2.21; 95% CI, 1.53-3.20; *P* < .001) and COPD (HR, 8.16; 95% CI, 4.55-14.64; *P* < .001) compared with nonsmoking (Table II). Increased incidence of COPD was also associated with male sex (HR, 2.19; 95% CI, 1.25-3.82; *P* = .01) and older baseline age (HR, 1.07 per year; 95% CI, 1.04-1.10; *P* < .001). The gut microbiome score was associated with increased incidence of both asthma (HR, 1.44 per SD; 95% CI, 1.23-1.67; *P* < .001) and COPD (HR, 1.39 per SD; 95% CI, 1.30-1.49; *P* < .001). In sensitivity analysis that additionally accounted for income and education levels, the findings of higher predictive capacity in C-index of the gut microbiome score than other individual risk factors further held for both asthma and COPD (see Table E3 in this article’s Online Repository at www.jacionline.org), and higher income and high education level were associated with lower risk of COPD (HR, 0.63, 95% CI, 0.53-0.76, *P* < .001, and HR, 0.3, 95% CI, 0.14-0.67, *P* = .003, respectively).

**Table II:** Association of risk factors separately and jointly for incident asthma and COPD

Covariate	Asthma				COPD			
	Univariable		Multivariable		Univariable		Multivariable	
	HR (95% CI)	*P* value	HR (95% CI)	*P* value	HR (95% CI)	*P* value	HR (95% CI)	*P* value
Sex: male	0.71 (0.49-1.03)	.07	0.67 (0.46-0.97)	.03	2.19 (1.25-3.82)	.01	1.35 (0.76-2.4)	.31
Baseline age (y)	0.99 (0.98-1.01)	.28	1.00 (0.98-1.01)	.75	1.07 (1.04-1.1)	<.001	1.1 (1.07-1.13)	<.001
BMI (kg/m^2^)	1.02 (0.99-1.06)	.22	1.03 (0.99-1.07)	.13	1.02 (0.97-1.08)	.48	0.99 (0.92-1.06)	.8
Smoking (yes)	2.21 (1.53-3.2)	<.001	2.06 (1.4-3.03)	<.001	8.16 (4.55-14.64)	<.001	11.07 (5.81-21.09)	<.001
Gut microbiome	1.44 (1.23-1.67)	<.001	1.34 (1.15-1.57)	<.001	1.39 (1.3-1.49)	<.001	1.18 (1.08-1.29)	<.001

Gut microbiome score is represented as microbiome-based predictions per SD. All analyses were performed in the validation set.

### Integrated prediction models of the gut microbiome and conventional risk factors

When integrating risk factors and gut microbiome score, the Cox model for asthma showed that current smoking status and gut microbiome were associated with higher risk (HR, 2.06, 95% CI, 1.40-3.03, *P* < .001, and HR, 1.34 per SD, 95% CI, 1.15-1.57, *P* < .001, respectively), and male sex was associated with lower risk (HR, 0.67, 95% CI, 0.46-0.97, *P* = .03), whereas there were no associations for baseline age and BMI at a statistical significance level (Table II). For COPD, baseline age, current smoking status, and gut microbiome score were statistically significant predictors (HR, 1.1 per year, 95% CI, 1.07-1.13, *P* < .001; HR, 11.07, 95% CI, 5.81-21.09, *P* < .001; and HR, 1.18 per SD, 95% CI, 1.08-1.29, *P* < .001, respectively). Although consistent with the individual predictive power of the gut microbiome score, the multivariable Cox model showed that the risk associated with current smokers at baseline was significantly greater than that for other risk factors for COPD. Sensitivity analyses additionally adjusting the Cox model for income and education level confirmed similar estimates of effects of sex, age, BMI, smoking, and the gut microbiome score in the combined model (see Table E3 in this article’s Online Repository at www.jacionline.org), whereas no statistical significance was detected for income and education.

**Fig 3:**
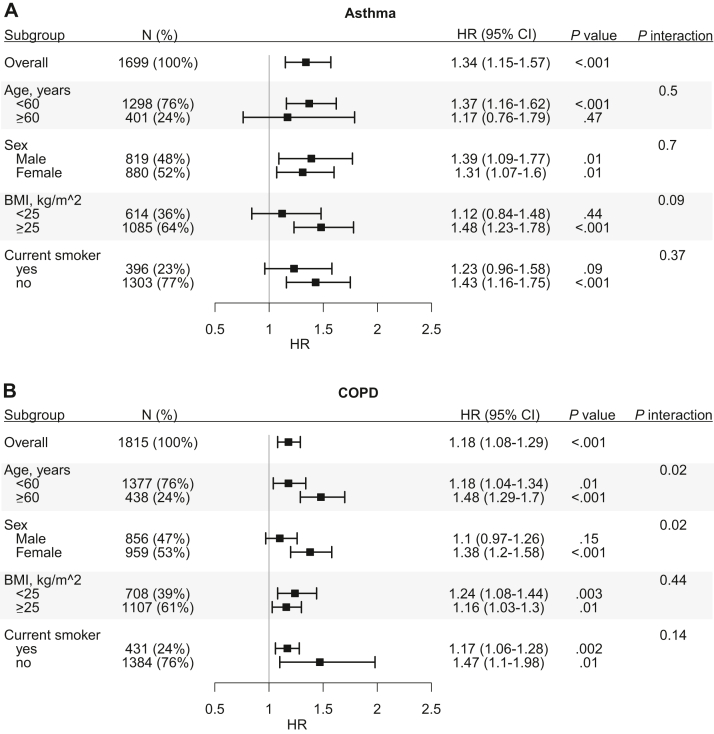
Subgroup analyses for (**A**) incident asthma or (**B**) COPD. Cox models were applied to test for interactions between gut microbiome and patient characteristic subgroups. Points and error bars represent HRs per SD and 95% CIs of gut microbiome score across subgroups.

In subgroup analyses, the gut microbiome score association patterns were generally consistent with those above (Fig 3). For COPD, where current smoking status had a relatively large HR, the gut microbiome score was independently associated with incident COPD in both current smokers and nonsmokers. In individuals who indicated past smoking but who were not current smokers at survey (n = 414), we found that the gut microbiome score was not associated with incident COPD (HR, 1.22 per SD; 95% CI, 0.89-1.68; *P* = .22) but that, in individuals who reported never smoking (n = 970), there was an association with incident COPD (HR, 1.40 per SD; 95% CI, 1.02-1.91; *P* = .04). Finally, in COPD, we observed evidence for statistical interactions of the gut microbiome score with age and sex (Fig 3).

**Fig 4:**
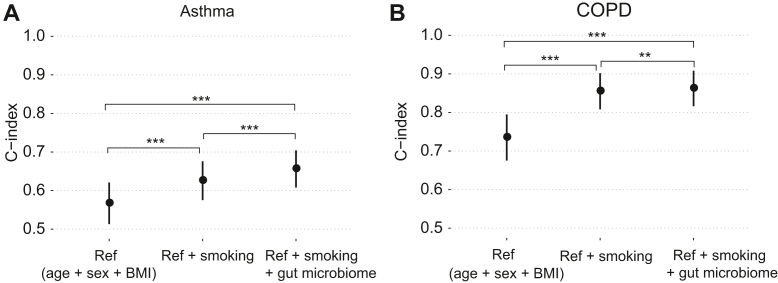
Predictive capacity of integrated models for (**A**) incident asthma and (**B**) COPD. “Ref” is a reference model that jointly considers age, sex, and BMI. Points and error bars represent the C-indices and 95% CIs. Analysis of deviance based on the log partial likelihood, ∗∗*P* < .01; ∗∗∗*P* < .001.

The integrated models showed improved predictive capacity for both incident asthma and COPD (Fig 4). For asthma, a reference model of age, sex, and BMI yielded C-index of 0.567; addition of smoking status and then gut microbiome score increased the C-index further to 0.626 and 0.656, respectively. For COPD, the reference model of age, sex, and BMI yielded C-index of 0.735; addition of smoking status and then gut microbiome score increased the C-index further to 0.855 and 0.862, respectively.

## Discussion

In this prospective study, we investigated the association and predictive capacity of the gut microbiome for future chronic respiratory diseases, asthma and COPD, in adults using shotgun metagenomics. We demonstrated that the gut microbiome is associated with incident asthma and COPD and evaluated the relative contributions of traditional risk factors and a gut microbiome score. We then constructed integrated risk models that maximized predictive performance. Taken together, our findings indicate that the gut microbiome is a potentially substantive biomarker with clinical validity for both asthma and COPD.

The gut and lung microbial communities, although residing in distal sites, are dominated by broadly similar bacterial phyla, including Firmicutes and Bacteroidetes, but differ in local compositions and total microbial biomass.^11^ Some of our findings are relevant to previous microbial studies of the respiratory tract. For example, *Haemophilus* and *Streptococcus* have been previously found to be positively associated with respiratory illnesses in the airways.^18^^,^^48^^,^^49^ In our gut microbiome samples, we also found positive associations between *Streptococcus* and incident asthma; however, we found that multiple *Haemophilus spp.* were negatively associated with incident COPD. An increased abundance of *Pseudomonas spp.* from the airway microbiome was previously reported in COPD exacerbations^50^^,^^51^ and impaired pulmonary function.^52^^,^^53^ Consistent with this, we found positive associations of the *Pseudomonas*, *Pseudomonas_A*, and *Pseudomonas_E* genera (all part of *Pseudomonas* according to the NCBI taxonomy) with incident asthma and COPD. These findings support the emerging evidence of possible functional links between the respiratory tract and the gastrointestinal tract; however, the underlying mechanisms by which microorganisms between the sites may interact remain unclear.^54^^,^^55^

Despite increasing recognition of the existence of gut-lung cross-talk, the role of the gut microbiota in respiratory disease has been primarily studied in children. Its relevance in adults has been unclear. Previous studies have demonstrated that the early-life gut microbial alteration and maturation patterns influence the risk of asthma development in childhood.^22^^,^^23^^,^^56^ In our data, we found that higher abundances of *Escherichia,*^31^ *Enterococcus*, *Clostridium*, *Veillonella*, and *B fragilis* were associated with increased incidence of asthma in adulthood, consistent with that observed for childhood asthma.^22^^,^^57^^,^^58^ In contrast to previous findings showing that the relative abundances of *Faecalibacterium*, *Roseburia*, and *Flavonifractor* were decreased in childhood asthma,^22^^,^^57^ we found positive associations with adult-onset asthma. We confirmed previous findings that increased abundances of *Clostridium* and *Eggerthella lenta* in the adult gut microbiome were associated with asthma.^27^ The relationship between the gut microbiome and COPD is even less understood. A recent study reported that *Streptococcus sp000187445* was enriched in patients with COPD and was correlated with reduced lung function,^30^ which was also confirmed by a positive association with incident COPD in our study.

Regarding consideration of causality in observational studies, it is challenging to determine whether the composition of the gut microbiome is a cause or consequence of respiratory disease. In this respect, one strength of our study was the use of baseline gut microbiome and incident disease systematically identified through EHRs. The follow-up using EHRs was nearly complete in all samples (except for the small number of participants who moved abroad permanently). Using machine learning models, we found that the baseline gut microbiome had moderate predictive capacities in distinguishing incident cases from noncases for asthma and COPD, suggesting that there are detectable changes in the gut microbiome antecedent to the onset of symptomatic disease. This does not confirm causality or eliminate other possibilities. For example, disease-associated host changes and gut microbial alteration may influence each other and operate simultaneously.^54^ We also showed that the association between gut microbiome–based predictions and incident asthma or COPD was largely independent of age, sex, BMI, and smoking, all of which can influence susceptibility to respiratory diseases.^59^, ^60^, ^61^, ^62^ Moreover, interactions of gut microbiome by sex and age were found for COPD with relatively weak signals, suggesting different impact of gut microbiome on age and sex groups, consistent with findings in other settings.^63^, ^64^, ^65^

Importantly, our study affirms the large body of evidence that smoking is associated with respiratory illness, especially COPD. Despite many ways to characterize the smoking phenotype, we found that individuals who reported being current smokers were at high risk of future asthma and COPD. The association between smoking and gut microbiota is well established, and smoking cessation has been shown to have profound, putatively causal effects on the gut microbiome.^66^ Our results show that, particularly for COPD, the gut microbiome is both a substantial independent predictor of future disease and that its predictive power is partially explained by smoking behavior. As such, our findings are both consistent with previous studies and take us a step closer to delineating which and to what extent particular gut microbial taxa sit along the causal path from smoking behavior to future asthma and COPD. For the latter, larger prospective studies will be necessary but population-scale gut microbiome and e-health studies are underway. There are other traditional risk factors that could be investigated in future studies, such as family history, environmental pollutants, exposure to allergens or irritants, and other lifestyles. Family history of asthma and/or allergy has been linked to increased risk of childhood asthma in particular, and the impact of paternal asthma continues to young adulthood.^67^, ^68^, ^69^ Although previous studies have suggested that the impact of family history of asthma and allergy decreases with age and paternal allergic disease is not associated with late-onset asthma with a cutoff age of 12 years,^70^^,^^71^ the effect of family history on adult-onset asthma could be further explored in the future.

There are limitations of the present study. First, despite a relatively large sample size, our study was enrolled from a single European country (Finland), and the generalizability of the findings to other geographically and culturally distinct settings will require further investigation in external cohorts with baseline gut microbiome and long-term respiratory disease data. Furthermore, only 1 time point of the gut microbiome was sampled per individual, which did not allow for dynamic or temporal assessment of gut microbiome alterations along with incident disease onset. Changes in diet and environmental exposures (apart from smoking) can induce changes in gut microbiota and should be considered in future studies. Limitations also concern the disease phenotyping in the present study, where incident cases were identified by a combination of EHRs of diagnosis codes, medicine purchases, and insurance reimbursements (see the Methods section). This might be subject to possible misclassification of borderline cases due to incorrect diagnostic labeling, particularly overlabeling of asthma. For example, patients with COPD might be mislabeled as asthma due to smoking-related stigma of COPD and better medication reimbursements of asthma. Inaccurate diagnosis may also be attributed to the asthma-COPD overlap syndrome characterized by coexistence of clinical features of both asthma and COPD.^72^ Future studies should consider differential diagnostic characteristics of asthma, COPD, and potentially a third group of asthma-COPD overlap syndrome for improved disease phenotyping.^73^, ^74^, ^75^ Although the asthma and COPD phenotypes can be difficult to diagnose or indeed overlap in some individuals, our study takes a pragmatic approach and future clinical cohorts may be necessary to precisely quantify disease-specific effects. Finally, although formal lung function test results (FEV_1_, FVC) may further improve prediction, it was not feasible to perform whole-scale clinical examination of airflow obstruction at the population level. Regardless, our study demonstrates that future exploration of the influence of the gut microbiome in severity and progression of asthma and COPD is warranted, and may lead to further clinically significant findings.

Our study supports the role of gut microbiome in adult respiratory disease and as potential biomarkers that might aid in risk profiling of asthma and COPD. The underlying mechanisms and causal links by which gut microbiota influence the lung, and vice versa, remain to be established.

**Key message**

• The gut microbiome features are associated with incident COPD and adult-onset asthma.
• The gut microbiome is potentially a predictive biomarker for primary prevention of asthma and COPD.
