# Supplementary material for: The gut microbiome is a significant risk factor for future chronic lung disease
Source: J Allergy Clin Immunol. 2023 Apr;151(4):943–52. doi: 10.1016/j.jaci.2022.12.810 (PMC10109092; doi:10.1016/j.jaci.2022.12.810)
Supplement: Supplementary data [file mmc1.docx]

**Fig S1. A-B,** Prediction of incident asthma and COPD using microbial features at different taxonomic levels individually and in combination. Models were developed in 70% of samples and tested in the rest 30% of samples. **C-D,** Prediction of incident asthma and COPD using gut microbial features at different taxonomy levels using 10 resampled data partitions. Error bars represent mean and standard deviation.
