# Supplementary material for: The gut microbiome is a significant risk factor for future chronic lung disease
Source: J Allergy Clin Immunol. 2023 Apr;151(4):943–52. doi: 10.1016/j.jaci.2022.12.810 (PMC10109092; doi:10.1016/j.jaci.2022.12.810)
Supplement: Supplementary Table S1 [file mmc2.pdf]

Table S1. Taxa associated with incident asthma by Cox models. HR, hazard ratio; CI, confidence interval. The Benjamini-Hochberg procedure was applied to each taoxnomic level for FDR correction. The Benjamini-Yekutieli procedure was applied across all taxonomic levels.

Taxonomic rank	Bacteria	beta	HR (95% CI)	FDR	BY
P	Firmicutes_A	0.298146	1.35 (1.11-1.63)	0.020735	0.10673
P	Proteobacteria	0.240248	1.27 (1.13-1.43)	0.003931	0.009682
P	Bacteroidota	0.206477	1.23 (1.1-1.38)	0.005576	0.025317
P	Firmicutes_C	0.164516	1.18 (1.08-1.29)	0.005576	0.027385
P	Verrucomicrobiota	-0.13046	0.88 (0.82-0.94)	0.005728	0.034513
C	Clostridia	0.315143	1.37 (1.13-1.66)	0.017253	0.069147
C	Gammaproteobacteria	0.232111	1.26 (1.14-1.4)	0.000722	0.002185
C	Bacteroidia	0.209153	1.23 (1.1-1.38)	0.004912	0.020833
C	Negativicutes	0.166563	1.18 (1.08-1.29)	0.004912	0.022664
C	Lentisphaeria	-0.15053	0.86 (0.8-0.92)	0.000896	0.00389
O	Paenibacillales	0.815776	2.26 (1.35-3.78)	0.016403	0.092439
O	Pseudomonadales	0.716295	2.05 (1.42-2.95)	0.003703	0.012369
O	Methylococcales	0.699232	2.01 (1.43-2.82)	0.002124	0.006876
O	Oscillospirales	0.370789	1.45 (1.18-1.78)	0.005413	0.026588
O	Eubacteriales	0.364085	1.44 (1.15-1.8)	0.016403	0.079448
O	Ectothiorhodospirales	0.355807	1.43 (1.11-1.84)	0.040189	0.206187
O	Brevibacillales	0.318097	1.37 (1.12-1.68)	0.016403	0.102693
O	Opitutales	-0.21701	0.8 (0.71-0.92)	0.012765	0.061201
O	Lachnospirales	0.205404	1.23 (1.08-1.4)	0.016403	0.087882
O	Burkholderiales	0.200708	1.22 (1.1-1.36)	0.005413	0.024508
O	Bacteroidales	0.198524	1.22 (1.1-1.36)	0.005413	0.020833
O	Erysipelotrichales	0.188323	1.21 (1.08-1.35)	0.008924	0.042727
O	Lactobacillales	0.159144	1.17 (1.05-1.3)	0.02495	0.142923
O	taxa4C28d_15	-0.14558	0.86 (0.81-0.93)	0.002124	0.005102
O	Victivallales	-0.14272	0.87 (0.81-0.93)	0.001955	0.002855
O	Christensenellales	-0.12818	0.88 (0.82-0.94)	0.005413	0.024619
O	RF39	-0.08909	0.91 (0.86-0.97)	0.016403	0.102103
O	ML615J_28	-0.0792	0.92 (0.88-0.97)	0.016403	0.099156
F	Paenibacillaceae	0.809685	2.25 (1.58-3.19)	0.000187	0.001573
F	Cellvibrionaceae	0.660231	1.94 (1.51-2.49)	6.02E-05	0.000187
F	Methylomonadaceae	0.646639	1.91 (1.48-2.46)	6.02E-05	0.000286
F	Rhodobacteraceae	0.599796	1.82 (1.28-2.58)	0.005303	0.048449
F	Pseudomonadaceae	0.597565	1.82 (1.41-2.34)	0.000176	0.001092
F	Halomonadaceae	0.584888	1.79 (1.32-2.44)	0.002148	0.017917
F	Aeromonadaceae	0.57987	1.79 (1.41-2.26)	9.62E-05	0.000557
F	Desulfitobacteriaceae	0.564425	1.76 (1.36-2.28)	0.000414	0.003307
F	Bacillaceae_C	0.490655	1.63 (1.32-2.02)	0.000181	0.001387

F	Fibrobacteraceae	0.475742	1.61 (1.25-2.08)	0.002437	0.021587
F	Oleiphilaceae	0.467646	1.6 (1.22-2.09)	0.004836	0.041794
F	Acetobacteraceae	0.466788	1.59 (1.14-2.22)	0.018335	0.208722
F	Xanthobacteraceae	0.466559	1.59 (1.16-2.19)	0.013208	0.152656
F	Xanthomonadaceae	0.464928	1.59 (1.19-2.13)	0.008261	0.087882
F	Caulobacteraceae	0.460409	1.58 (1.19-2.1)	0.00758	0.077096
F	Streptomycetaceae	0.456345	1.58 (1.19-2.09)	0.007982	0.082154
F	Beijerinckiaceae	0.440614	1.55 (1.15-2.1)	0.013645	0.157868
F	Sphingomonadaceae	0.440262	1.55 (1.12-2.16)	0.024692	0.284066
F	Micromonosporaceae	0.437732	1.55 (1.18-2.03)	0.008199	0.085978
F	Bacillaceae	0.430292	1.54 (1.21-1.95)	0.003113	0.026588
F	Anaerolineaceae	0.426005	1.53 (1.16-2.01)	0.010662	0.108815
F	Rhizobiaceae	0.425988	1.53 (1.09-2.15)	0.033714	0.40465
F	Micrococcaceae	0.425463	1.53 (1.22-1.91)	0.002148	0.017508
F	Hymenobacteraceae	0.422592	1.53 (1.19-1.96)	0.006143	0.058788
F	Acidobacteriaceae	0.417894	1.52 (1.13-2.05)	0.018984	0.216221
F	Planococcaceae	0.415532	1.52 (1.17-1.96)	0.007982	0.082154
F	Alteromonadaceae	0.414196	1.51 (1.19-1.92)	0.005046	0.046085
F	Bacillaceae_A	0.399149	1.49 (1.16-1.91)	0.008199	0.086235
F	Mycobacteriaceae	0.389762	1.48 (1.13-1.93)	0.014474	0.169946
F	Alcanivoracaceae	0.389531	1.48 (1.18-1.85)	0.004936	0.043618
F	Chromobacteriaceae	0.376934	1.46 (1.12-1.89)	0.014474	0.169816
F	Stappiaceae	0.369168	1.45 (1.08-1.93)	0.030604	0.355554
F	Hyphomonadaceae	0.369079	1.45 (1.09-1.92)	0.028625	0.322783
F	Streptosporangiaceae	0.365662	1.44 (1.1-1.88)	0.021771	0.248774
F	Brevibacillaceae	0.36504	1.44 (1.21-1.72)	0.001011	0.007368
F	Eubacteriaceae	0.364543	1.44 (1.19-1.74)	0.00191	0.013895
F	Vibrionaceae	0.357123	1.43 (1.12-1.82)	0.01386	0.161501
F	Pseudonocardiaceae	0.355359	1.43 (1.07-1.89)	0.033714	0.401726
F	Rhodanobacteraceae	0.354504	1.43 (1.09-1.86)	0.024381	0.280385
F	Sedimentibacteraceae	0.353178	1.42 (1.15-1.76)	0.006965	0.069525
F	Bacillaceae_D	0.34327	1.41 (1.13-1.76)	0.011731	0.120762
F	Spirosomaceae	0.339016	1.4 (1.1-1.79)	0.019172	0.220845
F	Azospirillaceae	0.336227	1.4 (1.08-1.81)	0.028768	0.332576
F	Cyanobiaceae	0.324958	1.38 (1.07-1.79)	0.032419	0.382174
F	Nostocaceae	0.322094	1.38 (1.12-1.7)	0.011731	0.123957
F	Sphingobacteriaceae	0.318	1.37 (1.11-1.7)	0.013104	0.148885
F	Chitinophagaceae	0.316904	1.37 (1.07-1.77)	0.033714	0.402934
F	Neisseriaceae	0.313774	1.37 (1.1-1.7)	0.01386	0.162553
F	Moraxellaceae	0.313752	1.37 (1.11-1.68)	0.012004	0.12903
F	UBA10450	0.31003	1.36 (1.05-1.77)	0.040913	0.502648
F	Geodermatophilaceae	0.309743	1.36 (1.08-1.72)	0.024381	0.279839
F	Cyclobacteriaceae	0.309382	1.36 (1.07-1.73)	0.028625	0.324942

F	Shewanellaceae	0.296367	1.34 (1.06-1.71)	0.036076	0.437392
F	Nocardioidaceae	0.290819	1.34 (1.05-1.71)	0.042997	0.531295
F	Bacillaceae_G	0.288987	1.34 (1.08-1.65)	0.02422	0.27453
F	Oscillospiraceae	0.288521	1.33 (1.13-1.58)	0.005804	0.0549
F	Amphibacillaceae	0.284256	1.33 (1.05-1.68)	0.040913	0.502191
F	Weeksellaceae	0.275906	1.32 (1.05-1.65)	0.037015	0.450914
F	Staphylococcaceae	0.275681	1.32 (1.12-1.55)	0.006204	0.062083
F	Aerococcaceae	0.272243	1.31 (1.09-1.58)	0.012794	0.145575
F	Enterococcaceae	0.265529	1.3 (1.13-1.5)	0.002148	0.016946
F	Flavobacteriaceae	0.265421	1.3 (1.05-1.63)	0.040435	0.491491
F	Vallitaleaceae	0.258201	1.29 (1.08-1.55)	0.014585	0.172322
F	Bacillaceae_H	0.255242	1.29 (1.05-1.59)	0.035854	0.431482
F	Butyricicoccaceae	0.254005	1.29 (1.13-1.46)	0.001423	0.010601
F	Listeriaceae	0.244254	1.28 (1.04-1.57)	0.043706	0.542112
F	Bacteriovoracaceae	0.239222	1.27 (1.04-1.55)	0.042997	0.531295
F	Leptotrichiaceae	0.236864	1.27 (1.05-1.52)	0.030441	0.351465
F	P3	0.231896	1.26 (1.09-1.46)	0.010479	0.10673
F	Eggerthellaceae	0.228839	1.26 (1.14-1.39)	0.000181	0.001353
F	Campylobacteraceae	0.225052	1.25 (1.04-1.51)	0.042997	0.531295
F	Lachnospiraceae	0.211907	1.24 (1.1-1.39)	0.003113	0.027385
F	Ruminococcaceae	0.206832	1.23 (1.09-1.39)	0.006204	0.062817
F	CAG_272	-0.20139	0.82 (0.74-0.9)	0.001215	0.008879
F	UBA1390	0.20087	1.22 (1.12-1.34)	0.000249	0.002161
F	Treponemataceae	0.197959	1.22 (1.04-1.42)	0.031484	0.369308
F	Burkholderiaceae	0.188857	1.21 (1.1-1.33)	0.001423	0.010741
F	Erysipelatoclostridiaceae	0.18324	1.2 (1.1-1.31)	0.000414	0.003146
F	Propionibacteriaceae	0.176607	1.19 (1.06-1.34)	0.012035	0.134368
F	Bacteroidaceae	0.173841	1.19 (1.09-1.29)	0.001008	0.006881
F	CAG_826	-0.17106	0.84 (0.74-0.96)	0.030953	0.360338
F	CAG_138	-0.16516	0.85 (0.79-0.91)	0.000146	0.000855
F	Streptococcaceae	0.157606	1.17 (1.07-1.28)	0.002921	0.024743
F	Actinomycetaceae	0.151527	1.16 (1.04-1.31)	0.028625	0.322734
F	UBA1750	-0.1498	0.86 (0.78-0.95)	0.010479	0.106575
F	Clostridiaceae	0.149184	1.16 (1.03-1.31)	0.033714	0.397412
F	Erysipelotrichaceae	0.148595	1.16 (1.05-1.28)	0.013208	0.152939
F	Peptostreptococcaceae	0.146595	1.16 (1.05-1.28)	0.012004	0.127572
F	CAG_382	-0.12958	0.88 (0.8-0.96)	0.017685	0.200105
F	Lactobacillaceae	0.119436	1.13 (1.02-1.24)	0.034179	0.411995
F	CAG_727	-0.11449	0.89 (0.83-0.95)	0.006183	0.060583
F	Megasphaeraceae	0.111131	1.12 (1.04-1.2)	0.012035	0.132464
F	Victivallaceae	-0.09941	0.91 (0.86-0.95)	0.002293	0.019572
F	Enterobacteriaceae	0.099364	1.1 (1.04-1.18)	0.011087	0.114237
F	CAG_917	-0.09869	0.91 (0.85-0.96)	0.00758	0.077096

F	UBA1829	-0.09237	0.91 (0.87-0.96)	0.002148	0.016611
F	CAG_1000	-0.08694	0.92 (0.87-0.97)	0.012064	0.136483
F	DTU072	-0.0838	0.92 (0.88-0.97)	0.004936	0.044304
F	Muribaculaceae	-0.07957	0.92 (0.87-0.98)	0.028768	0.329686
F	Veillonellaceae	0.075838	1.08 (1.01-1.15)	0.039318	0.475514
F	CAG_314	-0.07544	0.93 (0.89-0.97)	0.004301	0.036983
F	CAG_508	-0.07233	0.93 (0.88-0.99)	0.040435	0.490102
F	CAG_313	-0.06683	0.94 (0.9-0.98)	0.011731	0.12126
F	CAG_302	-0.0659	0.94 (0.89-0.98)	0.020786	0.239094
F	Acidaminococcaceae	0.064051	1.07 (1.01-1.13)	0.042909	0.523906
F	CAG_449	-0.05887	0.94 (0.91-0.97)	0.003347	0.029249
F	CAG_312	-0.05506	0.95 (0.92-0.97)	0.002437	0.021587
F	UBA644	-0.05068	0.95 (0.92-0.98)	0.005804	0.0549
F	CAG_552	-0.0483	0.95 (0.92-0.98)	0.012035	0.134853
F	UBA1820	-0.04696	0.95 (0.93-0.98)	0.011731	0.122919
F	QAMH01	-0.04188	0.96 (0.93-0.99)	0.012794	0.145575
G	Achromobacter	0.565144	1.76 (1.4-2.21)	4.06E-05	0.000488
G	Pseudomonas_E	0.548176	1.73 (1.41-2.13)	1.21E-05	0.000154
G	Bradyrhizobium	0.520607	1.68 (1.3-2.18)	0.000883	0.009194
G	Streptomyces	0.514379	1.67 (1.3-2.15)	0.000775	0.007904
G	Aeromonas	0.492525	1.64 (1.33-2.01)	7.12E-05	0.00086
G	Paenibacillus	0.481326	1.62 (1.29-2.02)	0.000368	0.003817
G	Stenotrophomonas	0.481041	1.62 (1.3-2.02)	0.000345	0.003531
G	Mesorhizobium	0.452758	1.57 (1.21-2.04)	0.003849	0.040892
G	Mycolicibacterium	0.444251	1.56 (1.23-1.98)	0.002226	0.022664
G	Halomonas	0.439369	1.55 (1.22-1.98)	0.002708	0.028703
G	Desulfosporosinus	0.434272	1.54 (1.27-1.87)	0.000227	0.002161
G	Xanthomonas	0.432269	1.54 (1.23-1.92)	0.001328	0.013895
G	Rhizobium	0.420395	1.52 (1.15-2.02)	0.013172	0.14809
G	Methylobacterium	0.419378	1.52 (1.19-1.94)	0.004279	0.044527
G	CAG_791	0.416958	1.52 (1.22-1.88)	0.001328	0.013683
G	Brevundimonas	0.408903	1.51 (1.2-1.88)	0.002484	0.025955
G	Terracidiphilus	0.386563	1.47 (1.16-1.86)	0.006436	0.069147
G	Anaerofilum	0.382029	1.47 (1.3-1.66)	5.40E-07	8.43E-06
G	Micromonospora	0.38191	1.47 (1.16-1.86)	0.007838	0.085342
G	Serratia	0.374696	1.45 (1.21-1.75)	0.000845	0.008678
G	Massilia	0.372958	1.45 (1.18-1.79)	0.003572	0.037704
G	Hymenobacter	0.372868	1.45 (1.19-1.78)	0.002226	0.022771
G	Bacillus	0.366837	1.44 (1.2-1.73)	0.000888	0.009298
G	Cupriavidus	0.366317	1.44 (1.14-1.83)	0.010498	0.118344
G	Herbaspirillum	0.365654	1.44 (1.17-1.78)	0.0038	0.040244
G	Marinobacter	0.365104	1.44 (1.16-1.78)	0.004637	0.048408
G	Eubacterium_H	0.364346	1.44 (1.19-1.74)	0.001558	0.016611

G	Clostridium_R	0.364035	1.44 (1.22-1.7)	0.000306	0.002855
G	Deinococcus	0.362791	1.44 (1.14-1.81)	0.00887	0.099155
G	Janthinobacterium	0.354695	1.43 (1.14-1.78)	0.008198	0.089732
G	Nocardia	0.35348	1.42 (1.1-1.84)	0.021653	0.234971
G	Burkholderia	0.351613	1.42 (1.11-1.83)	0.019001	0.206417
G	Sedimentibacter	0.348677	1.42 (1.19-1.68)	0.000883	0.009096
G	Azospirillum	0.348622	1.42 (1.14-1.77)	0.00887	0.099155
G	Actinoplanes	0.348239	1.42 (1.13-1.77)	0.009719	0.108524
G	UBA9475	0.346166	1.41 (1.23-1.63)	4.63E-05	0.000557
G	UBA3855	0.34586	1.41 (1.14-1.75)	0.006752	0.074109
G	Corynebacterium	0.343998	1.41 (1.14-1.75)	0.008543	0.094952
G	UBA2856	0.342567	1.41 (1.15-1.72)	0.004637	0.048408
G	Paraburkholderia	0.342468	1.41 (1.11-1.79)	0.016031	0.177375
G	Agrobacterium	0.341908	1.41 (1.15-1.73)	0.00626	0.065335
G	Paenibacillus_C	0.341036	1.41 (1.13-1.76)	0.010498	0.118695
G	Fournierella	0.337751	1.4 (1.23-1.6)	2.57E-05	0.000304
G	UBA1033	0.330558	1.39 (1.13-1.71)	0.008198	0.090053
G	Mycobacterium	0.327308	1.39 (1.14-1.69)	0.006752	0.074109
G	Brevibacillus	0.326858	1.39 (1.11-1.73)	0.013649	0.152939
G	Paracoccus	0.320774	1.38 (1.13-1.68)	0.008099	0.087882
G	Roseomonas	0.319365	1.38 (1.13-1.67)	0.006752	0.074109
G	Pantoea	0.316873	1.37 (1.12-1.69)	0.010498	0.118695
G	Pseudoflavonifractor	0.315868	1.37 (1.21-1.55)	2.05E-05	0.000256
G	Sphingobium	0.315604	1.37 (1.07-1.76)	0.033426	0.364427
G	taxa14_2	0.314839	1.37 (1.2-1.56)	6.07E-05	0.000728
G	Lactobacillus_G	0.312253	1.37 (1.15-1.62)	0.002708	0.028569
G	Sphingomonas	0.311135	1.36 (1.05-1.78)	0.049069	0.531295
G	MS4	0.308439	1.36 (1.15-1.62)	0.00287	0.030315
G	Bosea	0.306541	1.36 (1.08-1.72)	0.028	0.307519
G	Paraburkholderia_B	0.304189	1.36 (1.1-1.67)	0.015243	0.169286
G	Rhodococcus	0.302644	1.35 (1.09-1.68)	0.021223	0.229571
G	Bacillus_W	0.300598	1.35 (1.13-1.61)	0.004521	0.046663
G	Butyricicoccus	0.294826	1.34 (1.2-1.5)	1.21E-05	0.000166
G	GCA_900066575	0.286488	1.33 (1.17-1.51)	0.00027	0.002522
G	Lachnoclostridium_A	0.284523	1.33 (1.21-1.46)	1.12E-06	1.74E-05
G	Kineothrix	0.283258	1.33 (1.15-1.53)	0.001215	0.0123
G	Fibrobacter	0.28297	1.33 (1.08-1.62)	0.019669	0.213634
G	Hungatella_A	0.282405	1.33 (1.19-1.48)	1.21E-05	0.000165
G	Allorhizobium	0.281549	1.33 (1.05-1.67)	0.041182	0.450914
G	Anaerocolumna	0.280938	1.32 (1.12-1.56)	0.004814	0.051064
G	UBA7182	0.278608	1.32 (1.17-1.5)	0.000293	0.002718
G	Caulobacter	0.276788	1.32 (1.06-1.65)	0.037656	0.407606
G	Bittarella	0.273461	1.31 (1.17-1.47)	7.12E-05	0.000855

G	Sphingobacterium	0.272123	1.31 (1.12-1.54)	0.004645	0.048908
G	Lachnoanaerobaculum	0.271709	1.31 (1.11-1.56)	0.008679	0.096866
G	Kocuria	0.271246	1.31 (1.12-1.54)	0.004645	0.048962
G	Clostridioides	0.270488	1.31 (1.16-1.48)	0.000242	0.002215
G	An200	0.269871	1.31 (1.18-1.45)	1.40E-05	0.000187
G	Mucilaginibacter	0.267719	1.31 (1.11-1.54)	0.006436	0.068901
G	Nocardiopsis	0.266434	1.31 (1.07-1.59)	0.026068	0.285175
G	Photobacterium	0.261286	1.3 (1.1-1.53)	0.009227	0.104162
G	Chryseobacterium	0.259674	1.3 (1.09-1.54)	0.012383	0.138592
G	Oribacterium	0.258169	1.29 (1.06-1.58)	0.029577	0.323475
G	UBA1213	0.254831	1.29 (1.05-1.58)	0.038333	0.419465
G	Acinetobacter	0.253312	1.29 (1.09-1.52)	0.010368	0.116368
G	Clostridium_E	0.252942	1.29 (1.07-1.54)	0.019738	0.215109
G	Anaerotruncus	0.252841	1.29 (1.13-1.47)	0.001328	0.013711
G	Bacillus_A	0.25225	1.29 (1.09-1.53)	0.013172	0.14809
G	Eubacterium_Q	0.251224	1.29 (1.09-1.52)	0.012672	0.142763
G	UBA3282	0.250747	1.28 (1.09-1.51)	0.009888	0.110568
G	Anaeromassilibacillus	0.250325	1.28 (1.18-1.4)	1.12E-06	1.74E-05
G	Lysinibacillus	0.248759	1.28 (1.08-1.52)	0.015985	0.176641
G	Spirillospora	0.248645	1.28 (1.04-1.58)	0.048196	0.523906
G	Lawsonibacter	0.248019	1.28 (1.17-1.4)	3.99E-06	5.75E-05
G	Butyrivibrio	0.24296	1.28 (1.06-1.53)	0.025958	0.28344
G	Eubacterium	0.241621	1.27 (1.11-1.46)	0.0029	0.03073
G	Pseudoalteromonas	0.241243	1.27 (1.07-1.51)	0.016972	0.186945
G	Vibrio	0.241087	1.27 (1.05-1.54)	0.032454	0.355554
G	Stomatobaculum	0.239927	1.27 (1.09-1.48)	0.008679	0.096866
G	Flavonifractor	0.238451	1.27 (1.17-1.37)	1.12E-06	1.57E-05
G	Actinomyces	0.237035	1.27 (1.12-1.43)	0.001558	0.016598
G	Listeria	0.236195	1.27 (1.07-1.49)	0.016031	0.177375
G	Gemmiger_A	0.236074	1.27 (1.1-1.46)	0.005118	0.054552
G	Neobitarella	0.234336	1.26 (1.13-1.41)	0.000497	0.005102
G	Flavobacterium	0.231117	1.26 (1.06-1.5)	0.027033	0.295998
G	Aquimarina	0.228237	1.26 (1.08-1.46)	0.012945	0.145139
G	Yersinia	0.228156	1.26 (1.07-1.47)	0.016113	0.178212
G	OEMR01	0.227729	1.26 (1.14-1.38)	5.59E-05	0.00065
G	Agathobaculum	0.224412	1.25 (1.13-1.39)	0.000413	0.004203
G	Massilimaliae	0.224408	1.25 (1.1-1.42)	0.003127	0.03337
G	Chitinophaga	0.219387	1.25 (1.04-1.49)	0.041625	0.456325
G	Bacillus_X	0.219239	1.25 (1.06-1.47)	0.026299	0.288397
G	Clostridium_J	0.218831	1.24 (1.07-1.44)	0.013278	0.148919
G	Marseille_P3106	0.217064	1.24 (1.1-1.4)	0.002376	0.024619
G	Pedobacter	0.216714	1.24 (1.05-1.47)	0.030432	0.333744
G	Psychrobacter	0.215797	1.24 (1.07-1.44)	0.013649	0.152939

G	Enterococcus	0.214785	1.24 (1.08-1.42)	0.007838	0.08498
G	Staphylococcus	0.214401	1.24 (1.05-1.47)	0.03404	0.371052
G	UBA2882	0.214106	1.24 (1.1-1.39)	0.002708	0.02879
G	Faecalicatena	0.213075	1.24 (1.14-1.34)	1.16E-05	0.000144
G	Massilioclostridium	0.208785	1.23 (1.12-1.36)	0.000368	0.003817
G	Bacillus_AA	0.207401	1.23 (1.03-1.47)	0.049069	0.531299
G	Campylobacter_D	0.206926	1.23 (1.04-1.45)	0.037678	0.408982
G	Absiella	0.206421	1.23 (1.12-1.35)	0.00027	0.002522
G	Clostridium_B	0.201409	1.22 (1.04-1.44)	0.040261	0.439731
G	Marseille_P4683	0.200275	1.22 (1.08-1.38)	0.007478	0.081937
G	Blautia_A	0.197855	1.22 (1.11-1.33)	0.000297	0.002789
G	Prochlorococcus_A	0.194949	1.22 (1.04-1.43)	0.042224	0.462125
G	Oscillibacter	0.194455	1.21 (1.1-1.34)	0.000774	0.007792
G	UC5_1_2E3	0.194226	1.21 (1.12-1.31)	5.20E-05	0.000614
G	CHH4_2	0.194191	1.21 (1.1-1.35)	0.001793	0.019015
G	Marvinbryantia	0.194073	1.21 (1.07-1.38)	0.011601	0.12986
G	Tyzzerella	0.192932	1.21 (1.13-1.3)	8.53E-06	0.00011
G	Massiliomicrobiota	0.190843	1.21 (1.13-1.3)	3.99E-06	5.68E-05
G	Lactonifactor	0.188343	1.21 (1.11-1.32)	0.000345	0.003526
G	Clostridium_Q	0.187056	1.21 (1.11-1.31)	0.000345	0.003518
G	Acutalibacter	0.184982	1.2 (1.12-1.3)	4.63E-05	0.000557
G	Dorea	0.182399	1.2 (1.09-1.33)	0.002708	0.028703
G	Acetivibrio_A	0.181368	1.2 (1.07-1.35)	0.009719	0.108524
G	Lutibacter	0.17877	1.2 (1.05-1.37)	0.026584	0.291151
G	Enterococcus_B	0.178465	1.2 (1.07-1.34)	0.009774	0.108815
G	UBA1405	0.176802	1.19 (1.05-1.36)	0.023814	0.257251
G	Emergencia	0.175918	1.19 (1.06-1.34)	0.012252	0.13648
G	Sellimonas	0.174986	1.19 (1.11-1.28)	7.12E-05	0.00086
G	Gordonibacter	0.17374	1.19 (1.1-1.29)	0.000338	0.0032
G	CAG_81	0.170251	1.19 (1.09-1.29)	0.000643	0.006499
G	Hungatella	0.16916	1.18 (1.08-1.29)	0.001761	0.018591
G	Salmonella	0.164137	1.18 (1.08-1.29)	0.002494	0.026164
G	CHKCI006	0.161614	1.18 (1.08-1.28)	0.00107	0.010754
G	Clostridium_P	0.16061	1.17 (1.04-1.32)	0.025479	0.277318
G	Eubacterium_I	0.158467	1.17 (1.08-1.27)	0.001328	0.013895
G	Streptococcus	0.157729	1.17 (1.08-1.27)	0.001275	0.012913
G	Ruthenibacterium	0.157426	1.17 (1.07-1.28)	0.002708	0.028825
G	Blautia	0.157024	1.17 (1.08-1.27)	0.001157	0.011706
G	Phil1	-0.15519	0.86 (0.79-0.92)	0.00081	0.008267
G	Anaerostipes	0.154779	1.17 (1.09-1.25)	0.000338	0.0032
G	UBA737	-0.15458	0.86 (0.78-0.94)	0.00453	0.046983
G	Clostridium_M	0.15433	1.17 (1.09-1.25)	0.000131	0.001368
G	Erysipelatoclostridium	0.154112	1.17 (1.09-1.25)	0.000338	0.003307

G	Faecalibacterium	0.15377	1.17 (1.06-1.28)	0.006436	0.069147
G	An172	0.152239	1.16 (1.06-1.28)	0.006436	0.068086
G	Rubneribacter	0.148705	1.16 (1.06-1.27)	0.007254	0.079328
G	Eubacterium_E	0.145584	1.16 (1.06-1.26)	0.006794	0.074965
G	CAG_603	0.143733	1.15 (1.03-1.3)	0.037766	0.41249
G	Lactobacillus	0.14372	1.15 (1.07-1.25)	0.002376	0.024619
G	Proteus	0.14277	1.15 (1.02-1.3)	0.046808	0.509281
G	CAG_488	-0.14207	0.87 (0.77-0.97)	0.037766	0.411995
G	Clostridium_A	0.137571	1.15 (1.06-1.24)	0.002376	0.024619
G	Provencibacterium	0.134018	1.14 (1.03-1.27)	0.030651	0.337551
G	Staphylococcus_A	0.132751	1.14 (1.05-1.24)	0.009227	0.104162
G	Phil12	0.132516	1.14 (1.05-1.24)	0.008118	0.088295
G	UBA1390	0.132491	1.14 (1.07-1.22)	0.000522	0.005428
G	Coprobacillus	0.132401	1.14 (1.07-1.22)	0.001377	0.014397
G	Clostridium_AI	0.13213	1.14 (1.03-1.27)	0.040155	0.437392
G	Negativibacillus	0.130618	1.14 (1.05-1.23)	0.006196	0.064081
G	Faecalitalea	0.127911	1.14 (1.04-1.24)	0.011566	0.12903
G	Eisenbergiella	0.126303	1.13 (1.03-1.24)	0.022242	0.241272
G	Bacteroides	0.125571	1.13 (1.06-1.21)	0.002189	0.022483
G	Eggerthella	0.120925	1.13 (1.07-1.19)	0.000384	0.003971
G	Coprococcus_B	0.118184	1.13 (1.04-1.22)	0.017147	0.188815
G	Phocea	0.117792	1.13 (1.03-1.23)	0.026068	0.284914
G	Klebsiella_A	0.116863	1.12 (1.03-1.22)	0.022242	0.241272
G	Eubacterium_G	0.11459	1.12 (1.04-1.21)	0.009169	0.102649
G	Pauljensenia	0.112823	1.12 (1.02-1.22)	0.036563	0.397104
G	Klebsiella	0.111107	1.12 (1.04-1.2)	0.012361	0.137939
G	UBA1375	0.110301	1.12 (1.04-1.2)	0.014467	0.16099
G	Clostridium	0.109938	1.12 (1.02-1.22)	0.037766	0.412747
G	Holdemania	0.108793	1.11 (1.04-1.2)	0.010771	0.121088
G	PeH17	-0.10773	0.9 (0.85-0.94)	0.000338	0.003307
G	Fusicatenibacter	0.104926	1.11 (1.03-1.2)	0.017188	0.189916
G	Roseburia	0.104804	1.11 (1.04-1.19)	0.012672	0.142763
G	CAG_841	-0.10463	0.9 (0.85-0.95)	0.00298	0.031696
G	Ruminococcus_F	-0.10288	0.9 (0.83-0.98)	0.030432	0.334173
G	Duncaniella	-0.10179	0.9 (0.85-0.96)	0.005978	0.062021
G	CAG_390	-0.10161	0.9 (0.83-0.98)	0.043578	0.478004
G	CAG_533	-0.09808	0.91 (0.86-0.96)	0.002066	0.021587
G	Adlercreutzia	0.0978	1.1 (1.04-1.17)	0.007374	0.080602
G	Megasphaera	0.092689	1.1 (1.02-1.18)	0.028	0.307519
G	CAG_1024	-0.09246	0.91 (0.87-0.96)	0.001412	0.014837
G	UBA9502	0.092365	1.1 (1.02-1.18)	0.031679	0.348239
G	CAG_273	-0.09189	0.91 (0.87-0.96)	0.001162	0.011821
G	UBA1685	-0.09121	0.91 (0.86-0.97)	0.011404	0.127495

G	Agathobacter	0.09102	1.1 (1.02-1.17)	0.02581	0.280663
G	CAG_448	-0.09051	0.91 (0.86-0.97)	0.013172	0.147706
G	UBA1081	-0.09029	0.91 (0.85-0.98)	0.041182	0.450914
G	Intestinibacter	0.084998	1.09 (1.03-1.15)	0.008915	0.099851
G	UBA3792	-0.08475	0.92 (0.87-0.97)	0.014969	0.165971
G	CAG_349	-0.08475	0.92 (0.87-0.97)	0.010498	0.118344
G	UBA738	-0.08286	0.92 (0.86-0.99)	0.047276	0.515756
G	Anaerotignum	0.082482	1.09 (1.01-1.16)	0.045792	0.500554
G	Propionibacterium	0.081308	1.08 (1.02-1.16)	0.036685	0.398949
G	CAG_475	-0.0808	0.92 (0.88-0.97)	0.006603	0.070911
G	Acidaminococcus	0.080169	1.08 (1.02-1.15)	0.022845	0.247692
G	UBA4717	-0.07996	0.92 (0.89-0.95)	6.51E-05	0.000805
G	CAG_353	-0.07585	0.93 (0.87-0.99)	0.047276	0.514935
G	Veillonella	0.074614	1.08 (1.02-1.14)	0.025958	0.28344
G	CAG_177	-0.07358	0.93 (0.88-0.98)	0.014892	0.164924
G	CAG_724	-0.07265	0.93 (0.89-0.97)	0.010152	0.113729
G	Firm_11	-0.07052	0.93 (0.88-0.99)	0.045336	0.494279
G	UBA11512	-0.06821	0.93 (0.9-0.97)	0.00626	0.065046
G	QANA01	-0.06773	0.93 (0.9-0.97)	0.002442	0.025317
G	Victivallis	-0.0677	0.93 (0.9-0.97)	0.006436	0.068901
G	UBA4951	-0.06739	0.93 (0.89-0.98)	0.026068	0.284125
G	CAG_793	-0.06695	0.94 (0.9-0.97)	0.000952	0.00973
G	CAG_1138	-0.0636	0.94 (0.91-0.97)	0.001328	0.013895
G	UBA1206	-0.06246	0.94 (0.9-0.98)	0.027448	0.29998
G	CAG_272	-0.06196	0.94 (0.9-0.99)	0.029872	0.327143
G	CAG_1782	-0.06158	0.94 (0.91-0.97)	0.002066	0.021587
G	UBA7597	-0.06115	0.94 (0.9-0.98)	0.024164	0.261867
G	CAG_1000	-0.06091	0.94 (0.91-0.97)	0.003889	0.041597
G	Escherichia	0.059736	1.06 (1.02-1.11)	0.02581	0.280795
G	CAG_180	0.059153	1.06 (1.02-1.11)	0.015542	0.171529
G	CAG_302	-0.05871	0.94 (0.9-0.99)	0.035702	0.387822
G	CAG_460	-0.05702	0.94 (0.91-0.99)	0.02546	0.276226
G	UBA1259	-0.05612	0.95 (0.91-0.98)	0.008543	0.094695
G	UBA11452	-0.05578	0.95 (0.92-0.97)	0.000685	0.006881
G	CAG_312	-0.05404	0.95 (0.92-0.98)	0.002642	0.027385
G	CAG_313	-0.0538	0.95 (0.91-0.98)	0.014892	0.164659
G	CAG_1435	-0.05335	0.95 (0.92-0.98)	0.007936	0.086235
G	Oxalobacter	-0.05272	0.95 (0.92-0.97)	0.000758	0.007577
G	CAG_449	-0.05267	0.95 (0.92-0.98)	0.001857	0.019685
G	CAG_492	-0.05209	0.95 (0.91-0.99)	0.03553	0.385892
G	CAG_354	-0.05173	0.95 (0.92-0.98)	0.014024	0.157152
G	UBA644	-0.04996	0.95 (0.92-0.98)	0.006619	0.071482
G	CAG_452	-0.04983	0.95 (0.92-0.99)	0.025222	0.273709

G	UBA4636	-0.0472	0.95 (0.92-0.98)	0.011116	0.12433
G	UBA3789	-0.04563	0.96 (0.92-0.99)	0.02458	0.266337
G	UBA1820	-0.04504	0.96 (0.93-0.99)	0.014892	0.164314
G	UBA11475	-0.04494	0.96 (0.93-0.98)	0.008543	0.094695
G	CAG_582	-0.0435	0.96 (0.93-0.98)	0.00632	0.066364
G	CAG_822	-0.04131	0.96 (0.93-0.99)	0.027847	0.305259
G	Libanicoccus	-0.04102	0.96 (0.93-0.99)	0.043292	0.475514
G	QAMH01	-0.04086	0.96 (0.93-0.99)	0.015985	0.176641
G	CAG_826	-0.04012	0.96 (0.93-0.99)	0.022055	0.239276
G	CAG_552	-0.03895	0.96 (0.94-0.99)	0.010771	0.121088
G	M3	-0.03883	0.96 (0.94-0.99)	0.009227	0.104113
G	CAG_877	-0.03626	0.96 (0.94-0.99)	0.02126	0.230749
G	UBA10677	-0.03548	0.97 (0.94-0.99)	0.042236	0.463489
G	CAG_878	-0.03372	0.97 (0.94-0.99)	0.045304	0.492635
G	CAG_698	-0.03264	0.97 (0.94-0.99)	0.048558	0.527991
S	Lawsonibacter_sp000177015	0.302128	1.35 (1.21-1.51)	7.39E-06	6.64E-05
S	Anaerofilum_sp002160015	0.294251	1.34 (1.21-1.49)	3.77E-06	3.78E-05
S	Flavonifractor_sp002159455	0.265839	1.3 (1.18-1.44)	1.90E-05	0.000172
S	Clostridium_Q_saccharolyticum	0.262744	1.3 (1.19-1.42)	1.16E-06	1.37E-05
S	Flavonifractor_sp002159265	0.262498	1.3 (1.18-1.43)	7.93E-06	7.49E-05
S	Flavonifractor_sp002161085	0.255601	1.29 (1.17-1.43)	3.25E-05	0.000298
S	Fournierella_massiliensis	0.251195	1.29 (1.16-1.43)	0.000121	0.001092
S	Oscillibacter_sp000403435	0.24837	1.28 (1.13-1.45)	0.000954	0.008415
S	An200_sp002160025	0.246668	1.28 (1.16-1.42)	6.90E-05	0.00063
S	Pseudoflavonifractor_capillosus	0.241321	1.27 (1.15-1.41)	9.27E-05	0.000822
S	Faecalibacterium_sp002160895	0.240679	1.27 (1.14-1.42)	0.00023	0.002185
S	Butyricicoccus_pullicaecorum	0.23573	1.27 (1.15-1.39)	6.26E-05	0.000557
S	Flavonifractor_sp002159175	0.233688	1.26 (1.14-1.4)	0.00023	0.002185
S	Faecalibacterium_sp002160915	0.231362	1.26 (1.13-1.4)	0.000451	0.003971
S	Agathobaculum_desmolans	0.229158	1.26 (1.14-1.39)	0.000186	0.001737

S	Lachnoclostridium_A_sp002160755	0.229102	1.26 (1.17-1.35)	8.44E-07	8.43E-06
S	UBA9475_sp002161675	0.227176	1.26 (1.12-1.4)	0.001042	0.009096
S	Blautia_sp002161285	0.227026	1.25 (1.15-1.37)	1.36E-05	0.000135
S	Angelakisella_massiliensis	0.226664	1.25 (1.12-1.41)	0.001381	0.012114
S	Faecalicatena_sp002160525	0.221502	1.25 (1.14-1.37)	9.59E-05	0.000855
S	Anaerotruncus_colihominis	0.221261	1.25 (1.13-1.38)	0.000305	0.002909
S	Fournierella_sp002161595	0.216726	1.24 (1.13-1.37)	0.000244	0.002316
S	Faecalicatena_sp002159505	0.216325	1.24 (1.15-1.34)	9.95E-06	9.79E-05
S	Bittarella_massiliensis	0.216062	1.24 (1.13-1.37)	0.00023	0.002185
S	Flavonifractor_sp900199495	0.214557	1.24 (1.14-1.34)	1.25E-05	0.000121
S	Fournierella_sp002160145	0.213513	1.24 (1.11-1.38)	0.00107	0.009351
S	Clostridioides_difficile	0.212689	1.24 (1.12-1.36)	0.000284	0.002718
S	Absiella_innocuum	0.209093	1.23 (1.13-1.34)	6.90E-05	0.000614
S	UBA7182_sp002160135	0.208052	1.23 (1.12-1.35)	0.000305	0.002909
S	Fournierella_sp002159185	0.205587	1.23 (1.11-1.36)	0.000895	0.00791
S	Sellimonas_sp002159995	0.204361	1.23 (1.11-1.35)	0.000458	0.004127
S	Faecalicatena_sp900120155	0.199812	1.22 (1.11-1.35)	0.000767	0.006876
S	Flavonifractor_sp000508885	0.199291	1.22 (1.14-1.31)	3.45E-06	3.26E-05
S	Oscillibacter_valericigenes	0.198837	1.22 (1.07-1.4)	0.022301	0.157152
S	Hungatella_A_hathewayi	0.1981	1.22 (1.11-1.34)	0.000385	0.003496
S	Anaeromassilibacillus_sp001305115	0.197807	1.22 (1.13-1.32)	2.82E-05	0.000256
S	Agathobaculum_sp900291975	0.196358	1.22 (1.11-1.33)	0.000262	0.002522
S	Neobitarella_massiliensis	0.195823	1.22 (1.11-1.34)	0.000871	0.007715
S	Blautia_A_sp002159835	0.195533	1.22 (1.13-1.31)	2.91E-05	0.000269
S	Dorea_sp000765215	0.195156	1.22 (1.13-1.31)	1.83E-05	0.000166
S	Erysipelatoclostridium_sp002160495	0.193688	1.21 (1.13-1.31)	2.53E-05	0.00022
S	Eubacterium_E_sp900016875	0.192953	1.21 (1.12-1.32)	0.000167	0.001546
S	Dorea_scindens	0.192156	1.21 (1.12-1.31)	6.04E-05	0.000556
S	CAG_81_sp900066055	0.191472	1.21 (1.1-1.33)	0.000716	0.006442
S	Gemmiger_A_sp002160955	0.190602	1.21 (1.08-1.36)	0.007776	0.060993
S	Dorea_phocaeense	0.189276	1.21 (1.11-1.31)	0.000228	0.002161

S	OEMR01_sp900199515	0.189267	1.21 (1.11-1.31)	0.000134	0.001231
S	Clostridium_beijerinckii	0.188274	1.21 (1.07-1.36)	0.014446	0.104162
S	UBA9475_sp002161235	0.188261	1.21 (1.09-1.33)	0.002495	0.020833
S	Oscillibacter_sp000436875	0.18809	1.21 (1.11-1.31)	0.000145	0.001353
S	Massiliomicrobiota_sp002160815	0.187797	1.21 (1.13-1.28)	1.28E-06	1.57E-05
S	Flavonifractor_plautii	0.187095	1.21 (1.12-1.29)	1.51E-05	0.000148
S	Dorea_sp002160985	0.187044	1.21 (1.12-1.3)	9.59E-05	0.000855
S	Acetatifactor_muris	0.186931	1.21 (1.09-1.33)	0.002676	0.022077
S	Clostridium_M_asparagiforme	0.186114	1.2 (1.11-1.3)	0.000139	0.001306
S	GCA_900066575_sp002160825	0.1861	1.2 (1.12-1.3)	4.05E-05	0.000369
S	Marseille_P3106_sp900169975	0.184746	1.2 (1.09-1.33)	0.003289	0.026588
S	Butyricicoccus_A_porcorum	0.183427	1.2 (1.08-1.34)	0.006094	0.048408
S	GCA_900066575_sp002160765	0.183417	1.2 (1.12-1.29)	2.82E-05	0.000256
S	Blautia_A_sp900316115	0.182699	1.2 (1.08-1.33)	0.00389	0.030834
S	Clostridium_F_botulinum	0.181347	1.2 (1.06-1.35)	0.01958	0.138592
S	Gemmiger_variabile	0.180479	1.2 (1.08-1.33)	0.005872	0.046663
S	Clostridium_Q_symbiosum	0.177326	1.19 (1.11-1.29)	0.000139	0.001306
S	Lawsonibacter_sp002161175	0.175293	1.19 (1.08-1.32)	0.005275	0.041794
S	Tyzzerella_nexilis	0.175163	1.19 (1.11-1.27)	2.65E-05	0.000236
S	Faecalicatena_contorta_B	0.175049	1.19 (1.1-1.29)	0.000215	0.002022
S	Faecalicatena_fissicatena	0.174543	1.19 (1.08-1.31)	0.004367	0.034735
S	Faecalicatena_sp002320245	0.172784	1.19 (1.09-1.3)	0.001395	0.012293
S	Clostridium_M_clostridioforme	0.172581	1.19 (1.11-1.28)	0.00011	0.000971
S	Dorea_faecis	0.17196	1.19 (1.12-1.26)	2.77E-06	2.42E-05
S	Ruminococcus_A_sp002361775	0.171808	1.19 (1.08-1.3)	0.002692	0.022483
S	Faecalicatena_sp000509105	0.171567	1.19 (1.06-1.33)	0.020257	0.143593
S	CHH4_2_sp002899675	0.171245	1.19 (1.08-1.3)	0.002066	0.017435
S	UBA2882_sp900317505	0.17123	1.19 (1.08-1.3)	0.003456	0.027689
S	Sellimonas_sp002161525	0.170253	1.19 (1.1-1.27)	9.59E-05	0.000855
S	UC5_1_2E3_sp001304875	0.169555	1.18 (1.1-1.27)	0.000113	0.001016

S	Marseille_P4683_sp900232885	0.16926	1.18 (1.07-1.31)	0.010611	0.079448
S	Clostridium_M_clostridioforme_A	0.168759	1.18 (1.1-1.28)	0.000254	0.002428
S	Blautia_A_sp000285855	0.168719	1.18 (1.07-1.31)	0.005833	0.046351
S	Acetivibrio_A_ethanolgignens	0.168579	1.18 (1.07-1.31)	0.006977	0.054552
S	Agathobaculum_butyriciproducens	0.168053	1.18 (1.08-1.29)	0.002692	0.022483
S	Faecalicatena_gnavus	0.167714	1.18 (1.12-1.25)	1.03E-06	1.08E-05
S	Faecalicatena_glycyrrhizinilyticum	0.166842	1.18 (1.1-1.27)	0.000159	0.001466
S	Massilimaliae_timonensis	0.166737	1.18 (1.08-1.29)	0.001518	0.013271
S	Blautia_sp900120295	0.166318	1.18 (1.1-1.26)	6.90E-05	0.00063
S	Blautia_A_wexlerae	0.166121	1.18 (1.1-1.26)	6.26E-05	0.000557
S	Lactonifactor_longoviformis	0.165356	1.18 (1.09-1.27)	0.000457	0.004049
S	taxa14_2_sp001940225	0.164911	1.18 (1.08-1.29)	0.003709	0.029249
S	Anaeromassilibacillus_sp002159845	0.164602	1.18 (1.1-1.26)	0.000103	0.000903
S	Faecalicatena_sp002161355	0.163418	1.18 (1.09-1.27)	0.000621	0.005462
S	Clostridium_M_sp001304855	0.162888	1.18 (1.08-1.28)	0.001353	0.011821
S	Enterococcus_B_faecium_B	0.161815	1.18 (1.07-1.29)	0.003885	0.03073
S	Eubacterium_I_sp900066595	0.1618	1.18 (1.07-1.29)	0.003805	0.030055
S	UBA1405_sp002305685	0.158758	1.17 (1.05-1.31)	0.023842	0.169946
S	Faecalicatena_sp000364245	0.158287	1.17 (1.06-1.29)	0.011165	0.083763
S	CAG_81_sp900066535	0.157667	1.17 (1.09-1.26)	0.000297	0.002854
S	Blautia_A_schinkii	0.156522	1.17 (1.07-1.28)	0.00511	0.04049
S	CAG_45_sp002299665	0.1558	1.17 (1.06-1.29)	0.013184	0.095953
S	Faecalibacterium_prausnitzii_K	0.154849	1.17 (1.08-1.26)	0.000831	0.007368
S	Marvinbryantia_formatexigens	0.154332	1.17 (1.06-1.28)	0.010976	0.082154
S	Oscillibacter_sp900066435	0.154066	1.17 (1.09-1.25)	0.000284	0.002718
S	Blautia_producta	0.152713	1.16 (1.08-1.26)	0.001318	0.011538
S	Massilioclostridium_coli	0.15265	1.16 (1.07-1.26)	0.002692	0.022634

S	Lawsonibacter_sp002160305	0.151355	1.16 (1.09-1.24)	0.000186	0.001737
S	Lawsonibacter_asaccharolyticus	0.151101	1.16 (1.09-1.24)	0.00023	0.002185
S	Blautia_sp003287895	0.150781	1.16 (1.09-1.24)	0.000228	0.002161
S	Tyzzerella_sp000411335	0.150766	1.16 (1.09-1.24)	9.27E-05	0.000822
S	Flavonifractor_sp002161215	0.150098	1.16 (1.07-1.26)	0.002676	0.022039
S	Hungatella_hathewayi	0.149176	1.16 (1.09-1.24)	0.000284	0.002718
S	Blautia_sp001304935	0.147659	1.16 (1.08-1.24)	0.000458	0.004122
S	Lawsonibacter_sp900066645	0.146569	1.16 (1.07-1.25)	0.001715	0.014771
S	Anaerotignum_lactatifermentans	0.145405	1.16 (1.08-1.24)	0.00043	0.003817
S	Ruminococcus_A_sp000432335	0.142167	1.15 (1.07-1.24)	0.002495	0.020833
S	Phil1_sp001940855	-0.14211	0.87 (0.8-0.94)	0.002727	0.022801
S	COE1_sp000403335	0.141759	1.15 (1.04-1.28)	0.039871	0.288397
S	Enterococcus_B_faecium	0.141729	1.15 (1.04-1.27)	0.024762	0.177737
S	Erysipelatoclostridium_ramosum	0.141197	1.15 (1.09-1.22)	6.04E-05	0.000556
S	Lawsonibacter_sp900066825	0.141134	1.15 (1.07-1.24)	0.00107	0.009373
S	CHKCI006_sp900018345	0.140927	1.15 (1.07-1.24)	0.001461	0.012786
S	An200_sp003268275	0.140828	1.15 (1.08-1.23)	0.000458	0.004096
S	Blautia_A_sp900066505	0.139279	1.15 (1.05-1.26)	0.022257	0.156641
S	An172_sp002160515	0.138817	1.15 (1.06-1.25)	0.00719	0.056487
S	Eubacterium_E_sp002161065	0.13798	1.15 (1.07-1.23)	0.001621	0.013895
S	Bariatricus_massiliensis	0.136692	1.15 (1.03-1.28)	0.049636	0.360338
S	Emergencia_sp900066695	0.136662	1.15 (1.05-1.25)	0.013305	0.097134
S	Clostridium_M_citroniae	0.136203	1.15 (1.07-1.23)	0.001132	0.009941
S	Faecalibacterium_prausnitzii_J	0.134087	1.14 (1.05-1.24)	0.011033	0.082517
S	Ruthenibacterium_lactatiformans	0.133772	1.14 (1.06-1.24)	0.005664	0.045013
S	Enterococcus_faecalis	0.133477	1.14 (1.04-1.25)	0.024762	0.177375
S	Lachnoclostridium_A_edouardi	0.132557	1.14 (1.04-1.25)	0.02358	0.168269
S	Blautia_A_obeum_B	0.132354	1.14 (1.04-1.25)	0.022818	0.162553
S	Acutalibacter_timonensis	0.131372	1.14 (1.04-1.25)	0.025454	0.183185
S	Eubacterium_I_ramulus	0.1312	1.14 (1.06-1.22)	0.002405	0.019972
S	Dorea_sp900312975	0.131027	1.14 (1.05-1.24)	0.016488	0.118831

S	Eisenbergiella_massiliensis	0.129426	1.14 (1.06-1.22)	0.004157	0.032947
S	Negativibacillus_massiliensis	0.129417	1.14 (1.04-1.25)	0.026083	0.188815
S	UBA1390_sp002305315	0.128587	1.14 (1.07-1.21)	0.00043	0.003817
S	Clostridium_A_leptum	0.12834	1.14 (1.06-1.22)	0.002676	0.022165
S	Faecalitalea_cylindroides	0.127352	1.14 (1.05-1.23)	0.010929	0.081673
S	Clostridium_M_bolteae	0.126421	1.13 (1.07-1.2)	0.000296	0.002817
S	Coprobacillus_cateniformis	0.126376	1.13 (1.06-1.21)	0.001364	0.011915
S	Blautia_hansenii	0.125916	1.13 (1.06-1.22)	0.003824	0.030312
S	Blautia_A_hydrogenotrophica	0.1258	1.13 (1.05-1.22)	0.00719	0.056574
S	Clostridium_M_sp000155435	0.12568	1.13 (1.07-1.2)	0.000673	0.005955
S	Provencibacterium_massiliense	0.125455	1.13 (1.03-1.24)	0.03274	0.239411
S	Massilioclostridium_methylpentosum	0.124632	1.13 (1.04-1.23)	0.017457	0.125887
S	Dorea_formicigenerans	0.12442	1.13 (1.04-1.23)	0.024762	0.177375
S	Anaerostipes_sp900066705	0.123659	1.13 (1.06-1.21)	0.002268	0.018943
S	Agathobacter_sp002474415	0.122821	1.13 (1.05-1.22)	0.007163	0.056006
S	Anaerostipes_hadrus	0.121839	1.13 (1.06-1.2)	0.001061	0.00922
S	Coprococcus_B_comes	0.121063	1.13 (1.04-1.22)	0.015059	0.108815
S	Sellimonas_intestinalis	0.120401	1.13 (1.07-1.19)	0.000121	0.001092
S	CAG_83_sp000435975	-0.12031	0.89 (0.82-0.96)	0.024762	0.177375
S	Blautia_A_sp900120195	0.119815	1.13 (1.03-1.23)	0.038883	0.28344
S	Gordonibacter_urolithinfaciens	0.119475	1.13 (1.06-1.2)	0.001642	0.014151
S	Acutalibacter_sp000435395	0.11841	1.13 (1.07-1.19)	0.00043	0.003817
S	Bifidobacterium_breve	0.116938	1.12 (1.05-1.2)	0.00433	0.034443
S	Clostridium_M_lavalense	0.115266	1.12 (1.05-1.2)	0.005375	0.042727
S	Anaerostipes_caccae	0.115051	1.12 (1.05-1.2)	0.006031	0.047924
S	Phocea_massiliensis	0.114985	1.12 (1.04-1.22)	0.02574	0.186073
S	Erysipelatoclostridium_spiroforme	0.112616	1.12 (1.06-1.18)	0.000284	0.00272
S	Hungatella_effluvii	0.1126	1.12 (1.04-1.2)	0.010554	0.0788
S	Blautia_sp000432195	0.112558	1.12 (1.05-1.19)	0.002698	0.022664
S	CAG_603_sp900314525	0.112393	1.12 (1.03-1.21)	0.024762	0.177737
S	Blautia_A_sp900066165	0.112007	1.12 (1.03-1.21)	0.029486	0.216221

S	Faecalibacterium_prausnitzii_D	0.111897	1.12 (1.04-1.2)	0.013305	0.097134
S	Faecalibacterium_sp003449675	0.11168	1.12 (1.03-1.21)	0.028103	0.206187
S	Absiella_sp000165065	0.111597	1.12 (1.05-1.19)	0.007163	0.055978
S	Dorea_sp900240315	0.111538	1.12 (1.05-1.19)	0.005006	0.039669
S	Faecalibacterium_prausnitzii_E	0.109769	1.12 (1.03-1.21)	0.032977	0.241272
S	UBA1375_sp002305795	0.109376	1.12 (1.04-1.2)	0.014755	0.10673
S	Absiella_dolichum	0.109334	1.12 (1.04-1.2)	0.020257	0.143593
S	Acutalibacter_sp000432995	0.108972	1.12 (1.03-1.21)	0.035544	0.261867
S	Anaerotignum_sp001304995	0.107061	1.11 (1.04-1.19)	0.010184	0.076232
S	Blautia_A_sp000433815	0.106005	1.11 (1.06-1.17)	0.000895	0.00791
S	Eubacterium_G_ventriosum	0.105966	1.11 (1.05-1.18)	0.002495	0.020833
S	Absiella_sp000163515	0.104798	1.11 (1.03-1.19)	0.020996	0.14809
S	Faecalicatena_sp001487105	0.10463	1.11 (1.03-1.19)	0.022818	0.162956
S	Clostridium_M_sp001517625	0.104429	1.11 (1.06-1.17)	0.000717	0.006478
S	Eggerthella_lenta	0.103692	1.11 (1.06-1.17)	0.0007	0.006229
S	Faecalicatena_torques	0.103613	1.11 (1.05-1.17)	0.001095	0.009682
S	Dorea_sp900066765	0.103157	1.11 (1.03-1.2)	0.04053	0.2939
S	Fusicatenibacter_saccharivorans	0.102743	1.11 (1.04-1.19)	0.019059	0.135732
S	Phil12_sp002633275	0.100965	1.11 (1.04-1.18)	0.016503	0.119307
S	Lactococcus_lactis	0.099569	1.1 (1.03-1.18)	0.022742	0.161558
S	UBA9502_sp003506385	0.098455	1.1 (1.03-1.18)	0.02555	0.184289
S	Coprobacter_secundus	0.098151	1.1 (1.03-1.18)	0.027207	0.199857
S	Dorea_sp000509125	0.096678	1.1 (1.05-1.16)	0.001642	0.014139
S	Blautia_A_massiliensis	0.096031	1.1 (1.03-1.18)	0.022818	0.162605
S	COE1_sp003513705	0.095839	1.1 (1.02-1.18)	0.042201	0.307425
S	CAG_390_sp000437015	-0.09572	0.91 (0.86-0.96)	0.008648	0.066822
S	F23_B02_sp000431075	-0.09542	0.91 (0.87-0.95)	0.000704	0.006298
S	Bacteroides_fragilis_A	0.095032	1.1 (1.04-1.17)	0.010591	0.079321
S	Tyzzerella_sp000209385	0.094886	1.1 (1.04-1.17)	0.013736	0.099851
S	UBA737_sp002431945	-0.09166	0.91 (0.87-0.96)	0.005649	0.044749
S	Holdemania_filiformis	0.090696	1.09 (1.02-1.17)	0.033436	0.245476
S	CAG_170_sp003516765	-0.09058	0.91 (0.86-0.98)	0.032163	0.234971
S	PeH17_sp000435055	-0.08932	0.91 (0.87-0.96)	0.001138	0.010045

S	Oribacterium_sp900315665	-0.08778	0.92 (0.86-0.98)	0.036557	0.269394
S	Intestinibacter_bartlettii	0.087744	1.09 (1.04-1.15)	0.008007	0.062429
S	Faecalibacterium_prausnitzii_G	0.087604	1.09 (1.02-1.17)	0.049079	0.354444
S	CAG_1024_sp000432015	-0.08733	0.92 (0.87-0.96)	0.004307	0.034134
S	Clostridium_paraputrificum	0.087231	1.09 (1.02-1.17)	0.042187	0.306744
S	Bacteroides_caecimuris	0.087055	1.09 (1.03-1.16)	0.020748	0.146511
S	Roseburia_sp003483745	0.086909	1.09 (1.02-1.16)	0.033436	0.245612
S	Parabacteroides_sp900155425	0.086762	1.09 (1.03-1.16)	0.027207	0.199857
S	CAG_488_sp000434055	-0.0861	0.92 (0.87-0.96)	0.005275	0.041794
S	F23_B02_sp002472405	-0.08563	0.92 (0.86-0.98)	0.049623	0.359032
S	Agathobacter_faecis	0.084706	1.09 (1.03-1.15)	0.010976	0.082154
S	Clostridium_Q_sp000435655	0.083726	1.09 (1.03-1.15)	0.023842	0.169946
S	Bacteroides_acidifaciens	0.082734	1.09 (1.02-1.16)	0.040383	0.292271
S	Ruthenibacterium_sp003149955	0.082524	1.09 (1.02-1.16)	0.049636	0.360338
S	Clostridium_M_sp000431375	0.081829	1.09 (1.03-1.14)	0.016788	0.12126
S	Gordonibacter_pamelaeae	0.081046	1.08 (1.02-1.15)	0.042271	0.307519
S	Bacteroides_finegoldii	0.080594	1.08 (1.03-1.14)	0.021107	0.149021
S	UBA4717_sp002404395	-0.07925	0.92 (0.89-0.96)	0.000132	0.001208
S	Negativibacillus_sp000435195	0.078672	1.08 (1.03-1.14)	0.022301	0.157819
S	UBA1685_sp002320595	-0.07803	0.92 (0.87-0.98)	0.040903	0.297812
S	CAG_349_sp001940895	-0.07778	0.93 (0.88-0.98)	0.024555	0.175578
S	UBA1777_sp003150355	-0.07772	0.93 (0.87-0.98)	0.032438	0.237072
S	Bacteroides_xylanisolvens	0.076966	1.08 (1.03-1.14)	0.017707	0.127572
S	Dorea_sp000433535	0.076923	1.08 (1.02-1.14)	0.022728	0.16099
S	Bacteroides_rodentium	0.075827	1.08 (1.02-1.14)	0.026475	0.192411
S	Bifidobacterium_longum	0.075775	1.08 (1.02-1.14)	0.03714	0.273755
S	Bacteroides_A_sp002161565	0.074049	1.08 (1.02-1.14)	0.049079	0.354897
S	Streptococcus_parasanguinis_B	0.073897	1.08 (1.02-1.13)	0.025454	0.183185
S	Bacteroides_thetaiotaomicron	0.072947	1.08 (1.02-1.13)	0.029003	0.212387
S	Bacteroides_ovatus	0.07285	1.08 (1.02-1.13)	0.020996	0.14809
S	Escherichia_flexneri	0.072598	1.08 (1.02-1.13)	0.027201	0.198967

S	Massiliomicrobiota_timonensis	0.072325	1.08 (1.03-1.12)	0.008267	0.064262
S	Faecalicatena_sp002314255	0.071962	1.07 (1.02-1.14)	0.047761	0.344538
S	CAG_145_sp000435615	-0.07094	0.93 (0.89-0.98)	0.020257	0.143593
S	Propionibacterium_freudenreichii	0.070092	1.07 (1.02-1.13)	0.034133	0.250854
S	UBA737_sp002297415	-0.0693	0.93 (0.89-0.98)	0.027201	0.198822
S	Holdemania_sp900120005	0.06824	1.07 (1.02-1.13)	0.038065	0.279839
S	CAG_724_sp003524145	-0.06784	0.93 (0.89-0.98)	0.027201	0.198822
S	Rubneribacter_sp002159915	0.067457	1.07 (1.02-1.12)	0.038065	0.279839
S	Ruminococcus_D_sp000434695	-0.06734	0.93 (0.89-0.98)	0.04448	0.323475
S	PeH17_sp001940845	-0.06667	0.94 (0.9-0.98)	0.016457	0.118344
S	QANA01_sp003149735	-0.06567	0.94 (0.9-0.97)	0.005375	0.042727
S	UBA11512_sp003522145	-0.06539	0.94 (0.9-0.98)	0.014518	0.104573
S	Veillonella_parvula_A	0.065246	1.07 (1.02-1.11)	0.016256	0.116996
S	CAG_273_sp003507395	-0.06504	0.94 (0.91-0.97)	0.001621	0.013895
S	Firm_11_sp001604105	-0.06426	0.94 (0.9-0.98)	0.016488	0.118695
S	Escherichia_dysenteriae	0.063974	1.07 (1.02-1.11)	0.016488	0.118871
S	CAG_273_sp003534295	-0.0626	0.94 (0.91-0.97)	0.001164	0.010317
S	CAG_533_sp003150195	-0.06189	0.94 (0.9-0.98)	0.026475	0.192365
S	CAG_1138_sp000434675	-0.06172	0.94 (0.91-0.97)	0.002692	0.022501
S	UBA7597_sp002474405	-0.06115	0.94 (0.91-0.97)	0.003456	0.027722
S	CAG_177_sp003514385	-0.05613	0.95 (0.91-0.98)	0.029486	0.216221
S	CAG_841_sp000437375	-0.05553	0.95 (0.91-0.98)	0.022728	0.16099
S	Lactobacillus_gasseri_A	0.055352	1.06 (1.03-1.09)	0.001077	0.009485
S	Escherichia_coli_D	0.055239	1.06 (1.01-1.1)	0.034976	0.257251
S	UBA11452_sp003526375	-0.05499	0.95 (0.92-0.97)	0.00107	0.009351
S	CAG_1000_sp000434555	-0.05437	0.95 (0.92-0.97)	0.000815	0.007238
S	CAG_115_sp000432175	-0.05408	0.95 (0.91-0.98)	0.021547	0.152519
S	Victivallis_vadensis	-0.0529	0.95 (0.92-0.98)	0.005488	0.043618
S	CAG_180_sp000432435	0.052247	1.05 (1.02-1.09)	0.016525	0.120117
S	CAG_177_sp002451755	-0.05202	0.95 (0.91-0.99)	0.049344	0.356358
S	CAG_449_sp000432895	-0.05117	0.95 (0.92-0.98)	0.003456	0.027682
S	CAG_488_sp000434915	-0.05116	0.95 (0.92-0.98)	0.014694	0.106464
S	UBA1259_sp003523255	-0.05083	0.95 (0.92-0.98)	0.003209	0.025958
S	UBA1206_sp000433115	-0.05081	0.95 (0.92-0.98)	0.011506	0.085978
S	UBA644_sp002299265	-0.04865	0.95 (0.92-0.98)	0.013569	0.099155
S	CAG_354_sp001915925	-0.04863	0.95 (0.92-0.99)	0.032977	0.241272
S	Duncaniella_sp002494015	-0.04782	0.95 (0.93-0.98)	0.002334	0.019487
S	CAG_1782_sp002349735	-0.04776	0.95 (0.93-0.98)	0.002692	0.022634

S	Victivallis_sp002998355	-0.04702	0.95 (0.92-0.99)	0.039978	0.289667
S	CAG_1435_sp003537755	-0.04669	0.95 (0.93-0.98)	0.014755	0.10673
S	UBA4636_sp002405915	-0.04548	0.96 (0.93-0.99)	0.023895	0.170721
S	CAG_349_sp003539515	-0.04513	0.96 (0.93-0.99)	0.026083	0.188815
S	CAG_245_sp000434195	-0.04474	0.96 (0.93-0.98)	0.016525	0.119984
S	Paramuribaculum_intestinale	-0.04358	0.96 (0.93-0.99)	0.029424	0.215136
S	UBA11475_sp003538975	-0.0435	0.96 (0.93-0.99)	0.017437	0.125405
S	CAG_312_sp002437405	-0.04309	0.96 (0.93-0.98)	0.005275	0.041794
S	CAG_582_sp000435515	-0.04232	0.96 (0.93-0.98)	0.011899	0.087882
S	UBA1829_sp002405835	-0.0423	0.96 (0.93-0.99)	0.015513	0.111817
S	CAG_793_sp000433915	-0.04207	0.96 (0.93-0.99)	0.023176	0.164924
S	RC9_sp000433355	-0.04184	0.96 (0.93-0.99)	0.023281	0.165971
S	Methanobrevibacter_A_smithii	-0.04129	0.96 (0.93-0.98)	0.013184	0.096174
S	CAG_313_sp003539625	-0.04117	0.96 (0.93-0.99)	0.024183	0.172517
S	Butyricimonas_synergistica_A	-0.04104	0.96 (0.93-0.99)	0.015035	0.108537
S	Alloprevotella_tannerae	-0.04022	0.96 (0.94-0.98)	0.009308	0.07067
S	CAG_475_sp002449145	-0.04014	0.96 (0.93-0.99)	0.035544	0.261867
S	CAG_177_sp002438685	-0.0399	0.96 (0.94-0.99)	0.022742	0.161501
S	QAMH01_sp003149935	-0.0395	0.96 (0.93-0.99)	0.03274	0.239411
S	M3_sp001689445	-0.03913	0.96 (0.94-0.99)	0.016256	0.116894
S	Muribaculum_sp003150235	-0.03849	0.96 (0.94-0.99)	0.026402	0.19105
S	CAG_1435_sp000433775	-0.03822	0.96 (0.94-0.99)	0.021083	0.148885
S	CAG_533_sp002438065	-0.0378	0.96 (0.94-0.99)	0.040871	0.296456
S	CAG_552_sp000435495	-0.03769	0.96 (0.94-0.99)	0.022301	0.157819
S	CAG_492_sp000434335	-0.03685	0.96 (0.94-0.99)	0.047761	0.344538
S	UBA1820_sp003150615	-0.03664	0.96 (0.94-0.99)	0.014659	0.105899
S	CAG_877_sp000433455	-0.035	0.97 (0.94-0.99)	0.040903	0.297754
S	Oxalobacter_formigenes_A	-0.03422	0.97 (0.94-0.99)	0.034133	0.250662
S	CAG_533_sp000434495	-0.03315	0.97 (0.94-0.99)	0.030735	0.22531
S	Methanocorpusculum_sp001940805	-0.03205	0.97 (0.95-0.99)	0.039097	0.284125
S	UBA7173_sp002491305	-0.03168	0.97 (0.95-0.99)	0.04814	0.34792
S	CAG_312_sp001917305	-0.03102	0.97 (0.95-0.99)	0.044351	0.322734
