# Supplementary material for: The gut microbiome is a significant risk factor for future chronic lung disease
Source: J Allergy Clin Immunol. 2023 Apr;151(4):943–52. doi: 10.1016/j.jaci.2022.12.810 (PMC10109092; doi:10.1016/j.jaci.2022.12.810)
Supplement: Supplementary Table S2 [file mmc3.pdf]

Table S2. Taxa associated with incident COPD by Cox models. HR, hazard ratio; CI, confidence interval. The Benjamini-Hochberg procedure was applied to each taoxnomic level for FDR correction. The Benjamini-Yekutieli procedure was applied across all taxonomic levels.

Taxonomic rank	Bacteria	beta	HR (95% CI)	FDR	BY
P	Desulfuromonadota	0.973888	2.65 (1.37-5.13)	0.04246	0.239992
P	Patescibacteria	-0.96799	0.38 (0.19-0.74)	0.04246	0.266966
P	Bdellovibrionota	-0.82001	0.44 (0.26-0.76)	0.04246	0.199324
P	Thermotogota	-0.79251	0.45 (0.28-0.74)	0.04246	0.109343
P	Desulfobacterota_A	0.191082	1.21 (1.06-1.38)	0.04246	0.228607
C	Desulfuromonadia	0.991569	2.7 (1.42-5.1)	0.047924	0.164633
C	Paceibacteria	-0.93895	0.39 (0.21-0.74)	0.047924	0.255056
C	Thermotogae	-0.80642	0.45 (0.27-0.74)	0.047924	0.136918
C	Myxococcia	0.739668	2.1 (1.31-3.34)	0.047924	0.139986
C	Gracilibacteria	-0.67286	0.51 (0.32-0.8)	0.047924	0.227588
C	Ignavibacteria	-0.67065	0.51 (0.32-0.81)	0.047924	0.270064
C	Desulfovibrionia	0.190663	1.21 (1.07-1.37)	0.047924	0.216646
O	Streptomycetales	1.199541	3.32 (1.93-5.7)	0.000778	0.003855
O	Spirochaetales	1.148701	3.15 (1.82-5.45)	0.001207	0.007576
O	Rhodobacterales	1.111258	3.04 (1.51-6.12)	0.01409	0.139786
O	Caulobacterales	1.073958	2.93 (1.57-5.45)	0.007916	0.066631
O	Pseudomonadales	1.040731	2.83 (1.76-4.56)	0.000778	0.004833
O	Sphingomonadales	0.99487	2.7 (1.44-5.08)	0.01409	0.143875
O	Syntrophales	0.983331	2.67 (1.35-5.31)	0.0229	0.281392
O	Streptosporangiales	0.969871	2.64 (1.58-4.39)	0.003388	0.025759
O	Desulfobulbales	0.964559	2.62 (1.47-4.69)	0.010917	0.095856
O	Geobacterales	0.954562	2.6 (1.51-4.47)	0.007104	0.056404
O	Acidobacteriales	0.951874	2.59 (1.37-4.89)	0.017767	0.216646
O	Myxococcales	0.934353	2.55 (1.59-4.08)	0.002179	0.016376
O	Desulfuromonadales	0.913402	2.49 (1.46-4.26)	0.008597	0.075235
O	Azospirillales	0.910281	2.49 (1.52-4.07)	0.004588	0.035631
O	Acetobacterales	0.903642	2.47 (1.27-4.8)	0.034537	0.400585
O	Xanthomonadales	0.874021	2.4 (1.34-4.28)	0.017606	0.208721
O	Halobacteriales	0.855684	2.35 (1.35-4.09)	0.01511	0.171488
O	Mycobacteriales	0.848345	2.34 (1.55-3.53)	0.001414	0.010359
O	Ectothiorhodospirales	0.828199	2.29 (1.44-3.65)	0.006681	0.050762
O	Deinococcales	0.778368	2.18 (1.32-3.59)	0.01511	0.164633
O	Phycisphaerales	0.768678	2.16 (1.3-3.58)	0.017292	0.199324
O	Gemmatimonadales	0.679224	1.97 (1.25-3.12)	0.01833	0.23479
O	Bryobacterales	0.676031	1.97 (1.19-3.24)	0.034858	0.412524
O	Frankiales	0.652823	1.92 (1.23-2.99)	0.01833	0.236419
O	Rhodothermales	0.565765	1.76 (1.15-2.71)	0.039847	0.472618

O	Propionibacteriales	0.390902	1.48 (1.13-1.93)	0.01833	0.236419
O	Christensenellales	-0.28107	0.75 (0.67-0.86)	0.000778	0.003069
O	Erysipelotrichales	0.248771	1.28 (1.06-1.55)	0.043321	0.518901
O	taxa4C28d_15	-0.19839	0.82 (0.72-0.93)	0.01409	0.138601
O	Desulfovibrionales	0.195467	1.22 (1.07-1.38)	0.01409	0.147555
O	ML615J_28	-0.1516	0.86 (0.78-0.94)	0.011955	0.108859
O	RF39	-0.132	0.88 (0.79-0.97)	0.037107	0.44227
F	Streptomycetaceae	1.238152	3.45 (2.13-5.59)	0.000121	0.000346
F	Rhodobacteraceae	1.163807	3.2 (1.76-5.81)	0.001271	0.019544
F	Sphingomonadaceae	1.136496	3.12 (1.77-5.48)	0.000939	0.013685
F	Micromonosporaceae	1.111595	3.04 (1.91-4.83)	0.00013	0.001062
F	Caulobacteraceae	1.07272	2.92 (1.78-4.8)	0.000326	0.005221
F	Streptosporangiaceae	1.047537	2.85 (1.79-4.54)	0.000212	0.00298
F	Pseudonocardiaceae	1.042524	2.84 (1.74-4.62)	0.000372	0.005739
F	Dermatophilaceae	1.028097	2.8 (1.81-4.32)	0.00013	0.001306
F	Myxococcaceae	1.022396	2.78 (1.81-4.27)	0.00013	0.001197
F	Xanthobacteraceae	0.992625	2.7 (1.57-4.63)	0.002754	0.036413
F	Acetobacteraceae	0.992078	2.7 (1.52-4.79)	0.005053	0.067093
F	Devosiaceae	0.989782	2.69 (1.7-4.25)	0.000326	0.005221
F	Azospirillaceae	0.968751	2.63 (1.69-4.11)	0.000326	0.004878
F	Xanthomonadaceae	0.961077	2.61 (1.61-4.26)	0.001124	0.017042
F	Acidobacteriaceae	0.959774	2.61 (1.56-4.37)	0.00242	0.032404
F	Geodermatophilaceae	0.945423	2.57 (1.71-3.86)	0.00013	0.001701
F	Beijerinckiaceae	0.917187	2.5 (1.48-4.22)	0.004295	0.056404
F	Nocardioidaceae	0.881346	2.41 (1.56-3.73)	0.00091	0.012821
F	Halomonadaceae	0.879916	2.41 (1.42-4.08)	0.006811	0.090887
F	Pseudomonadaceae	0.877899	2.41 (1.66-3.48)	0.00013	0.001197
F	Chromobacteriaceae	0.82457	2.28 (1.45-3.58)	0.002909	0.03928
F	Microbacteriaceae	0.824505	2.28 (1.52-3.42)	0.00084	0.011516
F	GWC2_71_9	0.797875	2.22 (1.49-3.31)	0.00096	0.014083
F	Rhodocyclaceae	0.781991	2.19 (1.41-3.4)	0.003936	0.052154
F	Thermaceae	0.776659	2.17 (1.52-3.11)	0.000326	0.005174
F	Bryobacteraceae	0.767615	2.15 (1.37-3.39)	0.006317	0.081427
F	Haloferacaceae	0.761875	2.14 (1.36-3.38)	0.006811	0.089115
F	Frankiaceae	0.755266	2.13 (1.4-3.24)	0.003268	0.044461
F	Rhizobiaceae	0.751701	2.12 (1.2-3.74)	0.038621	0.451172
F	Mycobacteriaceae	0.751523	2.12 (1.51-2.98)	0.000318	0.004342
F	Micrococcaceae	0.742801	2.1 (1.52-2.9)	0.000156	0.002142
F	Koribacteraceae	0.741372	2.1 (1.29-3.41)	0.015529	0.190865
F	Aeromonadaceae	0.719567	2.05 (1.51-2.79)	0.00013	0.001583
F	Stappiaceae	0.708988	2.03 (1.24-3.33)	0.022321	0.273342
F	Deinococcaceae	0.670391	1.96 (1.25-3.07)	0.018259	0.228607
F	Hymenobacteraceae	0.668432	1.95 (1.28-2.96)	0.010177	0.131289

F	Geobacteraceae	0.655315	1.93 (1.25-2.96)	0.015529	0.193403
F	Hyphomonadaceae	0.652537	1.92 (1.18-3.12)	0.035579	0.415403
F	Alcanivoracaceae	0.607537	1.84 (1.25-2.71)	0.012498	0.15488
F	Pirellulaceae	0.604529	1.83 (1.22-2.75)	0.018259	0.228644
F	Haloarculaceae	0.595891	1.81 (1.21-2.71)	0.018259	0.228644
F	Natrialbaceae	0.584297	1.79 (1.2-2.68)	0.020444	0.255654
F	RAAP_2	0.575865	1.78 (1.2-2.64)	0.020444	0.256704
F	Oscillospiraceae	0.418383	1.52 (1.13-2.05)	0.026685	0.322714
F	Pasteurellaceae	-0.34685	0.71 (0.58-0.86)	0.003125	0.042113
F	UBA1390	0.297808	1.35 (1.16-1.57)	0.001063	0.015793
F	CAG_74	-0.28733	0.75 (0.67-0.85)	0.00013	0.001062
F	Propionibacteriaceae	0.285071	1.33 (1.09-1.62)	0.022817	0.28168
F	Caloramatoraceae	-0.27976	0.76 (0.61-0.93)	0.038621	0.45066
F	Erysipelotrichaceae	0.260573	1.3 (1.09-1.54)	0.016896	0.208721
F	CAG_727	-0.23431	0.79 (0.72-0.87)	0.00013	0.001621
F	Eggerthellaceae	0.230244	1.26 (1.06-1.5)	0.039786	0.466059
F	Desulfovibrionaceae	0.19304	1.21 (1.08-1.36)	0.007729	0.104212
F	CAG_302	-0.13958	0.87 (0.8-0.95)	0.007729	0.102744
F	CAG_313	-0.1346	0.87 (0.81-0.94)	0.005053	0.067206
F	Halobacillaceae_A	-0.08202	0.92 (0.87-0.98)	0.033582	0.396178
F	CAG_552	-0.07771	0.93 (0.88-0.98)	0.019751	0.243129
G	Streptomyces	1.012178	2.75 (1.8-4.21)	0.000106	0.001197
G	Micromonospora	0.936166	2.55 (1.71-3.79)	0.000122	0.001395
G	Nocardia	0.882035	2.42 (1.57-3.72)	0.001207	0.011164
G	Achromobacter	0.867617	2.38 (1.63-3.47)	0.00019	0.002142
G	Kitasatospora	0.842818	2.32 (1.57-3.43)	0.000549	0.005377
G	Mycolicibacterium	0.814613	2.26 (1.53-3.34)	0.000899	0.008442
G	Amycolatopsis	0.77837	2.18 (1.46-3.24)	0.002117	0.018214
G	Cupriavidus	0.758423	2.13 (1.43-3.2)	0.00322	0.029762
G	Brevundimonas	0.741469	2.1 (1.43-3.09)	0.00274	0.023381
G	Methylobacterium	0.731862	2.08 (1.37-3.16)	0.006241	0.060027
G	Devosia	0.702018	2.02 (1.36-3)	0.005541	0.052172
G	Variovorax	0.700341	2.01 (1.36-2.99)	0.005579	0.052887
G	Nocardiopsis	0.698645	2.01 (1.4-2.88)	0.00234	0.019922
G	Xanthomonas	0.68358	1.98 (1.34-2.92)	0.005875	0.055783
G	Halomonas	0.673273	1.96 (1.29-2.97)	0.01193	0.11857
G	Bradyrhizobium	0.669163	1.95 (1.24-3.06)	0.023747	0.228607
G	Nocardioides	0.663779	1.94 (1.32-2.86)	0.007502	0.072459
G	Novosphingobium	0.662038	1.94 (1.28-2.93)	0.013162	0.133178
G	Mesorhizobium	0.657164	1.93 (1.24-3.01)	0.024264	0.236419
G	Actinoplanes	0.657008	1.93 (1.31-2.84)	0.007998	0.079399
G	Rhizobium	0.645659	1.91 (1.18-3.08)	0.041906	0.422133
G	Azospirillum	0.641995	1.9 (1.31-2.77)	0.007523	0.073817

G	Herbaspirillum	0.633468	1.88 (1.31-2.71)	0.006193	0.05889
G	Microbacterium	0.631421	1.88 (1.33-2.65)	0.003865	0.037294
G	Sphingomonas	0.626357	1.87 (1.19-2.93)	0.033625	0.33387
G	Sphingobium	0.62312	1.86 (1.22-2.86)	0.025423	0.251293
G	Curtobacterium	0.620848	1.86 (1.29-2.68)	0.00758	0.075143
G	Gemmiger_A	0.613961	1.85 (1.47-2.33)	2.16E-05	0.000203
G	Stenotrophomonas	0.602204	1.83 (1.24-2.69)	0.017185	0.169626
G	Chromobacterium	0.600303	1.82 (1.25-2.65)	0.012677	0.12874
G	Allorhizobium	0.591633	1.81 (1.21-2.7)	0.024264	0.236419
G	Caulobacter	0.588315	1.8 (1.21-2.68)	0.023747	0.228644
G	Bosea	0.587932	1.8 (1.19-2.71)	0.029081	0.281392
G	Deinococcus	0.586266	1.8 (1.21-2.67)	0.024264	0.236419
G	Gordonia	0.585998	1.8 (1.22-2.65)	0.021049	0.203776
G	Pseudoflavonifractor	0.58389	1.79 (1.46-2.2)	9.54E-06	5.02E-05
G	Mycobacterium	0.577415	1.78 (1.3-2.45)	0.00434	0.042114
G	UBA9475	0.566687	1.76 (1.39-2.23)	9.63E-05	0.001087
G	Pseudomonas_A	0.566411	1.76 (1.21-2.56)	0.020773	0.200951
G	Pseudomonas_E	0.56635	1.76 (1.24-2.51)	0.012445	0.125333
G	Aeromonas	0.563784	1.76 (1.29-2.39)	0.003826	0.036413
G	Ruminococcus	-0.559	0.57 (0.4-0.81)	0.014577	0.144249
G	Spirillospora	0.549173	1.73 (1.19-2.51)	0.024264	0.236419
G	Lysobacter	0.54588	1.73 (1.2-2.49)	0.023747	0.228607
G	Sphingopyxis	0.541739	1.72 (1.17-2.52)	0.031497	0.306412
G	Fournierella	0.535998	1.71 (1.36-2.15)	0.000136	0.001621
G	Ralstonia	0.535707	1.71 (1.2-2.44)	0.021679	0.210042
G	Paraburkholderia_B	0.53403	1.71 (1.18-2.47)	0.027011	0.264948
G	Pseudonocardia	0.523963	1.69 (1.16-2.46)	0.033625	0.336348
G	Massilia	0.519979	1.68 (1.16-2.44)	0.033625	0.335888
G	Olsenella	0.487025	1.63 (1.27-2.08)	0.001983	0.016837
G	Anaerofilum	0.484102	1.62 (1.31-2.01)	0.000253	0.002879
G	UBA1405	0.475142	1.61 (1.28-2.02)	0.000836	0.007834
G	Hymenobacter	0.465397	1.59 (1.13-2.24)	0.039403	0.39798
G	Butyricicoccus	0.464207	1.59 (1.34-1.89)	2.16E-05	0.000182
G	Rhodococcus	0.46263	1.59 (1.2-2.1)	0.009657	0.095856
G	Marseille_P3106	0.458125	1.58 (1.3-1.92)	0.000136	0.001621
G	Provencibacterium	0.442547	1.56 (1.31-1.85)	3.14E-05	0.000326
G	RUG420	0.440102	1.55 (1.11-2.17)	0.046599	0.465668
G	Acutalibacter	0.438055	1.55 (1.38-1.74)	2.14E-10	6.70E-09
G	NK3B98	0.419808	1.52 (1.1-2.1)	0.048702	0.490813
G	Pseudomonas	0.411597	1.51 (1.21-1.89)	0.003826	0.036413
G	UBA7182	0.403662	1.5 (1.21-1.85)	0.003072	0.027946
G	Lawsonibacter	0.386908	1.47 (1.27-1.71)	3.12E-05	0.000296
G	An200	0.378078	1.46 (1.23-1.74)	0.00052	0.005221

G	Anaeromassilibacillus	0.368156	1.45 (1.25-1.67)	3.81E-05	0.000447
G	Anaerotruncus	0.367189	1.44 (1.16-1.8)	0.009436	0.093433
G	Merdibacter	0.359405	1.43 (1.23-1.66)	8.19E-05	0.000944
G	TF01_11	-0.35435	0.7 (0.61-0.81)	5.26E-05	0.000647
G	Neobitarella	0.347528	1.42 (1.18-1.7)	0.002975	0.026358
G	Lachnoclostridium_A	0.336109	1.4 (1.18-1.66)	0.001627	0.014083
G	Flavonifractor	0.333864	1.4 (1.22-1.59)	3.57E-05	0.000396
G	UC5_1_2E3	0.330574	1.39 (1.22-1.58)	3.48E-05	0.000362
G	OEMR01	0.328288	1.39 (1.19-1.62)	0.000755	0.007044
G	An172	0.327612	1.39 (1.19-1.62)	0.00072	0.006698
G	Eubacterium_R	-0.32525	0.72 (0.63-0.82)	6.69E-05	0.000802
G	CAG_95	-0.3204	0.73 (0.59-0.89)	0.013925	0.139856
G	Bittarella	0.318607	1.38 (1.13-1.67)	0.010042	0.099997
G	Hungatella_A	0.316368	1.37 (1.13-1.67)	0.011235	0.111599
G	GCA_900066135	-0.31354	0.73 (0.59-0.91)	0.027652	0.270064
G	Actinomyces	0.308879	1.36 (1.11-1.67)	0.022875	0.221039
G	Oscillibacter	0.305432	1.36 (1.15-1.6)	0.004151	0.040439
G	COE1	-0.29911	0.74 (0.62-0.88)	0.006585	0.063732
G	Traorella	0.29855	1.35 (1.09-1.66)	0.031497	0.305611
G	Coprococcus_A	-0.29098	0.75 (0.66-0.84)	8.19E-05	0.000952
G	Enorma	0.28871	1.33 (1.14-1.57)	0.00434	0.042581
G	Faecalicoccus	0.28534	1.33 (1.19-1.49)	3.14E-05	0.000326
G	Negativibacillus	0.276451	1.32 (1.16-1.49)	0.000377	0.004038
G	Succiniclasticum	0.272584	1.31 (1.12-1.54)	0.006838	0.066577
G	Romboutsia	-0.2724	0.76 (0.66-0.88)	0.002369	0.020125
G	UBA1375	0.261407	1.3 (1.11-1.51)	0.007502	0.072837
G	CHKCI006	0.258923	1.3 (1.13-1.49)	0.003503	0.03365
G	Massiliomicrobiota	0.257888	1.29 (1.15-1.46)	0.000507	0.005136
G	UBA11524	-0.25729	0.77 (0.7-0.85)	1.05E-05	6.56E-05
G	Lachnospira	-0.24414	0.78 (0.69-0.89)	0.002975	0.026196
G	Absiella	0.237894	1.27 (1.08-1.49)	0.024984	0.245274
G	CAG_353	-0.23503	0.79 (0.7-0.9)	0.003253	0.030655
G	Sellimonas	0.233445	1.26 (1.12-1.43)	0.003072	0.027507
G	UBA737	-0.23195	0.79 (0.69-0.92)	0.012445	0.124594
G	Eubacterium_F	-0.22167	0.8 (0.71-0.9)	0.004151	0.040439
G	Hungatella	0.2212	1.25 (1.07-1.46)	0.032784	0.321992
G	Bacteroides_F	-0.21972	0.8 (0.68-0.95)	0.047673	0.475379
G	An7	0.215437	1.24 (1.05-1.46)	0.048702	0.490813
G	Mailhella	0.215403	1.24 (1.11-1.39)	0.002975	0.025828
G	Faecalicatena	0.213852	1.24 (1.08-1.42)	0.015955	0.157388
G	ER4	-0.21189	0.81 (0.7-0.94)	0.027011	0.264948
G	Faecalitalea	0.207766	1.23 (1.06-1.42)	0.030455	0.29338
G	Haemophilus_D	-0.20519	0.81 (0.76-0.88)	1.48E-05	0.000103

G	UBA5920	-0.20244	0.82 (0.7-0.95)	0.046313	0.460373
G	Ruminococcus_C	-0.18925	0.83 (0.72-0.95)	0.041906	0.423662
G	KLE1615	-0.18456	0.83 (0.73-0.95)	0.029197	0.282694
G	UBA11774	-0.18325	0.83 (0.76-0.92)	0.002975	0.026358
G	UBA1390	0.179746	1.2 (1.07-1.34)	0.010933	0.108014
G	UBA9502	-0.17621	0.84 (0.74-0.95)	0.033381	0.328017
G	CAG_127	-0.17619	0.84 (0.73-0.96)	0.04473	0.45012
G	CAG_56	-0.17359	0.84 (0.75-0.95)	0.027011	0.264948
G	QAMM01	0.159419	1.17 (1.04-1.32)	0.041516	0.415403
G	Acidaminococcus	0.150518	1.16 (1.06-1.27)	0.009436	0.092804
G	CAG_302	-0.14979	0.86 (0.79-0.93)	0.003253	0.030993
G	Coprococcus	-0.14922	0.86 (0.77-0.96)	0.033625	0.335527
G	V9D3004	-0.14704	0.86 (0.77-0.96)	0.041396	0.412736
G	UBA1685	-0.14534	0.86 (0.78-0.96)	0.036385	0.368405
G	CAG_882	-0.14162	0.87 (0.79-0.95)	0.018264	0.179963
G	CAG_115	-0.13987	0.87 (0.79-0.96)	0.024364	0.23905
G	Caecibacter	0.124373	1.13 (1.03-1.25)	0.048702	0.490813
G	CAG_217	0.122168	1.13 (1.03-1.23)	0.033859	0.33987
G	Desulfovibrio	0.120214	1.13 (1.03-1.23)	0.044471	0.445121
G	QALS01	-0.11581	0.89 (0.83-0.95)	0.005413	0.050868
G	CAG_180	0.111926	1.12 (1.05-1.2)	0.008933	0.087939
G	CAG_313	-0.10657	0.9 (0.84-0.96)	0.007998	0.078836
G	CAG_288	-0.08528	0.92 (0.87-0.97)	0.011195	0.110212
G	UBA1259	-0.08176	0.92 (0.87-0.98)	0.035425	0.35727
G	Haemophilus_A	-0.07514	0.93 (0.88-0.98)	0.032212	0.315324
S	Flavonifractor_sp002159455	0.506249	1.66 (1.4-1.96)	7.20E-07	9.71E-06
S	Flavonifractor_sp002159175	0.473465	1.61 (1.35-1.91)	7.04E-06	9.12E-05
S	Flavonifractor_sp002159265	0.457998	1.58 (1.35-1.85)	1.60E-06	2.26E-05
S	Lawsonibacter_sp002161175	0.441851	1.56 (1.31-1.84)	2.53E-05	0.000285
S	Flavonifractor_sp002161085	0.429267	1.54 (1.3-1.81)	2.59E-05	0.000296
S	Faecalicatena_sp002160525	0.41187	1.51 (1.29-1.77)	3.08E-05	0.000326
S	An200_sp002160025	0.410713	1.51 (1.27-1.79)	8.99E-05	0.000897
S	GCA_900066575_sp002160825	0.389898	1.48 (1.3-1.67)	3.57E-07	4.49E-06
S	Gemmiger_A_sp002160955	0.387784	1.47 (1.21-1.79)	0.001721	0.013891

S	Flavonifractor_sp002161215	0.381152	1.46 (1.29-1.66)	7.49E-07	1.03E-05
S	Faecalicatena_sp002159505	0.379698	1.46 (1.28-1.67)	4.49E-06	6.06E-05
S	Pseudoflavonifractor_capillosus	0.376755	1.46 (1.23-1.73)	0.000571	0.004342
S	Faecalicatena_sp002161355	0.374113	1.45 (1.27-1.67)	7.04E-06	9.12E-05
S	Oscillibacter_sp000403435	0.371448	1.45 (1.18-1.78)	0.006294	0.048983
S	Faecalicatena_sp900120155	0.353734	1.42 (1.2-1.68)	0.000874	0.00688
S	UBA9475_sp002161235	0.352598	1.42 (1.2-1.69)	0.001469	0.011578
S	Dorea_sp900312975	0.348236	1.42 (1.23-1.63)	8.89E-05	0.000861
S	GCA_900066575_sp002160765	0.346118	1.41 (1.25-1.6)	3.82E-06	5.02E-05
S	Eubacterium_E_sp900016875	0.341271	1.41 (1.22-1.62)	8.99E-05	0.000897
S	Fournierella_massiliensis	0.340188	1.41 (1.17-1.69)	0.004519	0.035335
S	Lawsonibacter_sp002160305	0.335198	1.4 (1.26-1.55)	2.07E-07	2.45E-06
S	Lawsonibacter_sp000492175	0.334484	1.4 (1.13-1.73)	0.019734	0.142781
S	Butyricicoccus_pullicaecorum	0.333412	1.4 (1.19-1.63)	0.000868	0.006761
S	Provencibacterium_massiliense	0.331789	1.39 (1.19-1.63)	0.000728	0.005403
S	UBA1417_sp002305575	0.327053	1.39 (1.21-1.58)	7.82E-05	0.000724
S	UBA9475_sp002161675	0.323259	1.38 (1.14-1.68)	0.011583	0.087431
S	Dorea_phocaeense	0.321607	1.38 (1.2-1.59)	0.000268	0.002076
S	Lachnoclostridium_A_sp002160755	0.321203	1.38 (1.22-1.56)	3.00E-05	0.000326
S	Marseille_P3106_sp900169975	0.320157	1.38 (1.16-1.64)	0.004512	0.034822
S	Flavonifractor_sp900199495	0.317736	1.37 (1.2-1.58)	0.000239	0.001813
S	UBA1405_sp002305685	0.316516	1.37 (1.13-1.66)	0.01335	0.099131
S	Anaerofilum_sp002160015	0.31621	1.37 (1.14-1.65)	0.008401	0.064457
S	Acutalibacter_sp000435395	0.314729	1.37 (1.25-1.5)	2.19E-08	1.72E-07
S	Dorea_faecis	0.313161	1.37 (1.24-1.51)	1.08E-07	1.13E-06

S	Sellimonas_sp002161525	0.303015	1.35 (1.2-1.52)	3.13E-05	0.000335
S	Fournierella_sp002161595	0.301405	1.35 (1.14-1.6)	0.005806	0.044126
S	GCA_900066135_sp900066135	-0.29868	0.74 (0.6-0.91)	0.039245	0.266017
S	Coprococcus_A_catus	-0.29397	0.75 (0.66-0.84)	0.000112	0.001062
S	Faecalicatena_sp002314255	0.292188	1.34 (1.22-1.47)	3.87E-07	5.07E-06
S	Blautia_A_sp002159835	0.292131	1.34 (1.18-1.52)	0.00038	0.002898
S	Faecalibacterium_sp002160895	0.291014	1.34 (1.12-1.6)	0.016043	0.117953
S	Blautia_sp002161285	0.290398	1.34 (1.16-1.55)	0.001857	0.015087
S	CAG_81_sp900066785	-0.2885	0.75 (0.65-0.87)	0.002506	0.020125
S	Acutalibacter_sp000432995	0.288382	1.33 (1.17-1.53)	0.000737	0.005739
S	UBA7182_sp002160135	0.288275	1.33 (1.13-1.57)	0.006499	0.050868
S	COE1_sp001916965	-0.28827	0.75 (0.65-0.86)	0.000911	0.00717
S	TF01_11_sp001916135	-0.28371	0.75 (0.65-0.87)	0.001785	0.014279
S	Clostridium_Q_saccharolyticum	0.277766	1.32 (1.13-1.54)	0.006294	0.048983
S	Merdibacter_massiliensis	0.274702	1.32 (1.15-1.5)	0.001031	0.008034
S	Anaerotignum_lactatifermentans	0.274538	1.32 (1.18-1.47)	8.56E-05	0.000802
S	Dorea_sp000765215	0.271665	1.31 (1.15-1.5)	0.001086	0.008564
S	Dorea_sp002160985	0.270615	1.31 (1.15-1.5)	0.001721	0.013891
S	Fournierella_sp002160145	0.268547	1.31 (1.08-1.58)	0.042659	0.292386
S	Eubacterium_E_sp002161065	0.265633	1.3 (1.16-1.47)	0.000408	0.003069
S	UBA11524_sp000437595	-0.26308	0.77 (0.7-0.84)	3.88E-06	5.17E-05
S	Oscillibacter_sp000436875	0.262858	1.3 (1.13-1.5)	0.004594	0.035923
S	UC5_1_2E3_sp001304875	0.259186	1.3 (1.15-1.46)	0.000715	0.005297
S	Faecalicoccus_pleomorphus	0.255626	1.29 (1.16-1.43)	8.56E-05	0.000815
S	Faecalibacterium_prausnitzii_I	-0.25314	0.78 (0.67-0.9)	0.010102	0.075772
S	An172_sp002160515	0.251051	1.29 (1.12-1.48)	0.006294	0.04874

S	Anaeromassilibacillus_sp001305115	0.250629	1.28 (1.13-1.47)	0.003293	0.026358
S	Clostridium_M_sp001304855	0.249612	1.28 (1.11-1.48)	0.006875	0.05353
S	Neobitarella_massiliensis	0.244531	1.28 (1.09-1.5)	0.028546	0.203776
S	Massiliomicrobiota_sp002160815	0.244293	1.28 (1.14-1.43)	0.000715	0.005221
S	Sellimonas_sp002159995	0.242042	1.27 (1.08-1.51)	0.038681	0.262478
S	OEMR01_sp900199515	0.239619	1.27 (1.11-1.46)	0.00701	0.054871
S	Hungatella_A_hathewayi	0.23431	1.26 (1.07-1.49)	0.042659	0.292386
S	CAG_95_sp000438155	-0.23093	0.79 (0.68-0.92)	0.027883	0.199324
S	Lawsonibacter_asaccharolyticus	0.229667	1.26 (1.12-1.41)	0.001721	0.013748
S	Oscillibacter_sp900066435	0.226473	1.25 (1.11-1.41)	0.003132	0.025683
S	Megasphaera_sp900066485	0.225284	1.25 (1.09-1.44)	0.016781	0.123456
S	CAG_353_sp900066885	-0.22525	0.8 (0.71-0.9)	0.002506	0.020151
S	Faecalibacterium_prausnitzii_F	-0.22066	0.8 (0.71-0.91)	0.006207	0.047192
S	TF01_11_sp003524945	-0.21999	0.8 (0.72-0.9)	0.002466	0.019544
S	ER4_sp000765235	-0.21505	0.81 (0.7-0.92)	0.018515	0.135364
S	Absiella_innocuum	0.211229	1.24 (1.06-1.44)	0.04657	0.317924
S	Anaeromassilibacillus_sp002159845	0.208385	1.23 (1.09-1.39)	0.006756	0.052598
S	TF01_11_sp001414325	-0.20697	0.81 (0.72-0.92)	0.01128	0.08472
S	Erysipelatoclostridium_sp002160495	0.206913	1.23 (1.08-1.4)	0.019516	0.140567
S	Lachnospira_sp900316325	-0.2066	0.81 (0.74-0.9)	0.000733	0.005502
S	UBA1375_sp002305795	0.20652	1.23 (1.07-1.41)	0.028546	0.203663
S	Lachnospira_eligens_B	-0.20466	0.81 (0.73-0.91)	0.005302	0.040439
S	Anaerotruncus_sp900199635	0.20065	1.22 (1.07-1.39)	0.02525	0.179963
S	Flavonifractor_sp000508885	0.200034	1.22 (1.08-1.38)	0.015912	0.116448
S	Roseburia_hominis	-0.19979	0.82 (0.71-0.94)	0.037262	0.252091
S	TF01_11_sp003529475	-0.19811	0.82 (0.74-0.91)	0.001703	0.013515
S	CHKCI006_sp900018345	0.195056	1.22 (1.07-1.38)	0.019739	0.143463

S	UBA11774_sp003507655	-0.1903	0.83 (0.75-0.91)	0.002466	0.019544
S	Negativibacillus_sp000435195	0.187868	1.21 (1.11-1.32)	0.000715	0.005221
S	Blautia_A_sp000436615	-0.18562	0.83 (0.74-0.93)	0.010052	0.075235
S	Lachnospira_sp003537285	-0.18457	0.83 (0.77-0.9)	0.00048	0.003666
S	KLE1615_sp900066985	-0.18301	0.83 (0.73-0.95)	0.03983	0.270064
S	CAG_170_sp003516765	-0.18197	0.83 (0.75-0.93)	0.012965	0.095856
S	Eubacterium_R_sp000436835	-0.18121	0.83 (0.74-0.94)	0.034334	0.236419
S	UBA9502_sp003506385	-0.1803	0.84 (0.74-0.95)	0.041875	0.282694
S	Flavonifractor_plautii	0.180095	1.2 (1.06-1.35)	0.037262	0.252091
S	Mailhella_sp003150275	0.179433	1.2 (1.08-1.32)	0.005494	0.041963
S	CAG_56_sp900066615	-0.17694	0.84 (0.75-0.94)	0.024079	0.171488
S	Lachnospira_sp003451515	-0.17012	0.84 (0.77-0.92)	0.003045	0.024812
S	Hungatella_hathewayi	0.168067	1.18 (1.05-1.33)	0.044678	0.305611
S	Eubacterium_F_sp003491505	-0.1648	0.85 (0.77-0.93)	0.007911	0.060683
S	Lachnospira_sp000436535	-0.16355	0.85 (0.8-0.9)	1.60E-06	2.26E-05
S	TF01_11_sp003149875	-0.16288	0.85 (0.77-0.94)	0.019826	0.144224
S	Anaerostipes_hadrus_A	-0.16114	0.85 (0.78-0.93)	0.002823	0.022855
S	Coprococcus_eutactus	-0.16032	0.85 (0.76-0.95)	0.032162	0.227588
S	UBA737_sp002431945	-0.16028	0.85 (0.78-0.93)	0.002506	0.020088
S	Ruminococcus_C_callidus	-0.15912	0.85 (0.77-0.95)	0.032156	0.227379
S	Clostridium_Q_sp000435655	0.157768	1.17 (1.06-1.29)	0.017705	0.129281
S	F23_B02_sp002472405	-0.15739	0.85 (0.77-0.94)	0.019826	0.14427
S	CAG_103_sp000432375	-0.15558	0.86 (0.77-0.95)	0.034334	0.236419
S	Bacteroides_fluxus	0.154237	1.17 (1.05-1.3)	0.040942	0.278567
S	UBA1390_sp002305315	0.152727	1.17 (1.05-1.29)	0.032156	0.227379
S	CAG_45_sp000438375	-0.15041	0.86 (0.8-0.93)	0.002929	0.023706
S	Anaerotignum_sp001304995	0.150341	1.16 (1.04-1.29)	0.048493	0.331889
S	Lachnospira_sp000436475	-0.15015	0.86 (0.78-0.96)	0.040119	0.272714
S	UBA1777_sp003150355	-0.14955	0.86 (0.79-0.94)	0.009261	0.070409
S	CAG_145_sp000435615	-0.14893	0.86 (0.8-0.92)	0.000737	0.005739
S	UBA1685_sp002320595	-0.14374	0.87 (0.78-0.96)	0.04951	0.336348
S	Faecalicatena_torques	0.142489	1.15 (1.05-1.26)	0.01805	0.131882

S	Clostridium_sp000753455	0.136523	1.15 (1.06-1.24)	0.010665	0.08007
S	CAG_510_sp000434615	-0.13453	0.87 (0.8-0.96)	0.038681	0.262278
S	Rothia_mucilaginosa_A	0.131786	1.14 (1.07-1.22)	0.001046	0.008232
S	CAG_882_sp003486385	-0.1302	0.88 (0.81-0.96)	0.025953	0.185783
S	Collinsella_stercoris	0.129618	1.14 (1.04-1.24)	0.038681	0.261745
S	Romboutsia_timonensis	-0.12914	0.88 (0.82-0.94)	0.002466	0.019551
S	Eubacterium_R_sp000434995	-0.1237	0.88 (0.83-0.95)	0.005327	0.040439
S	Streptococcus_sp000187445	0.122911	1.13 (1.07-1.2)	0.000571	0.004326
S	CAG_115_sp000432175	-0.12268	0.88 (0.84-0.94)	0.000737	0.005739
S	QALS01_sp003150575	-0.11838	0.89 (0.83-0.95)	0.005511	0.042113
S	PeH17_sp001940845	-0.11788	0.89 (0.83-0.95)	0.008828	0.067093
S	CAG_273_sp000438355	0.117042	1.12 (1.06-1.2)	0.004519	0.035335
S	Clostridium_sp000435835	-0.11146	0.89 (0.85-0.94)	0.000584	0.004469
S	CAG_882_sp000435595	-0.1101	0.9 (0.83-0.97)	0.047521	0.323783
S	CAG_115_sp003531585	-0.1084	0.9 (0.84-0.96)	0.025955	0.1866
S	CAG_180_sp000432435	0.100886	1.11 (1.05-1.17)	0.006493	0.050762
S	Ruminococcus_C_sp000437255	-0.09832	0.91 (0.85-0.97)	0.038774	0.264164
S	Mogibacterium_sp002299625	0.095833	1.1 (1.03-1.18)	0.042659	0.292386
S	Haemophilus_D_sp001679485	-0.09393	0.91 (0.88-0.95)	8.56E-05	0.000802
S	Lactobacillus_gasseri_A	0.093417	1.1 (1.04-1.15)	0.003439	0.027507
S	Haemophilus_D_parainfluenzae	-0.09196	0.91 (0.87-0.95)	0.000715	0.005221
S	Veillonella_rogosae	-0.09188	0.91 (0.87-0.95)	0.001469	0.011661
S	Veillonella_dispar_A	-0.08995	0.91 (0.88-0.95)	0.000737	0.005739
S	CAG_288_sp000437395	-0.08745	0.92 (0.87-0.97)	0.012162	0.091019
S	Haemophilus_D_parainfluenzae_L	-0.08643	0.92 (0.88-0.95)	0.000305	0.002326
S	Streptococcus_thermophilus	-0.08611	0.92 (0.87-0.97)	0.014761	0.108859
S	Haemophilus_D_parainfluenzae_K	-0.08189	0.92 (0.89-0.96)	0.001363	0.010823
S	Haemophilus_D_parainfluenzae_M	-0.08127	0.92 (0.89-0.96)	0.001086	0.008627
S	CAG_302_sp000431795	-0.07835	0.92 (0.88-0.97)	0.01022	0.077048
S	Haemophilus_D_parainfluenzae_N	-0.07741	0.93 (0.89-0.96)	0.002506	0.019949

S	Haemophilus_D_sp001815355	-0.07632	0.93 (0.89-0.96)	0.001792	0.014449
S	Streptococcus_sp001587175	-0.07605	0.93 (0.89-0.97)	0.006499	0.050868
S	Pauljensenia_sp000278725	-0.07512	0.93 (0.89-0.97)	0.014342	0.106158
S	Haemophilus_D_parainfluenzae_A	-0.07277	0.93 (0.89-0.97)	0.006294	0.048932
S	CAG_826_sp000437235	-0.06738	0.93 (0.89-0.98)	0.030775	0.216646
S	Veillonella_infantium	-0.06651	0.94 (0.9-0.97)	0.014201	0.10461
S	Leuconostoc_pseudomesenteroides	-0.05949	0.94 (0.9-0.98)	0.03983	0.270064
S	Desulfovibrio_sp900319575	0.059191	1.06 (1.02-1.11)	0.048493	0.331889
S	Veillonella_parvula	-0.05719	0.94 (0.91-0.98)	0.042659	0.292386
