# Supplementary material for: The gut microbiome is a significant risk factor for future chronic lung disease
Source: J Allergy Clin Immunol. 2023 Apr;151(4):943–52. doi: 10.1016/j.jaci.2022.12.810 (PMC10109092; doi:10.1016/j.jaci.2022.12.810)
Supplement: Supplementary Table S3 [file mmc4.docx]

**Table S3. Sensitivity analysis accounting for income class and education level.** Association of risk factors was assessed separately and in combination for incident asthma and COPD in samples with complete data of variables. HR, hazard ratio; CI, confidence interval; BMI, body mass index; Gut microbiome score is represented as microbiome-based predictions per SD. All analyses were performed in the validation set.

|  | |  | **Asthma** | | | | | |  | **COPD** | | | | | |
| --- | --- | --- | --- | --- | --- | --- | --- | --- | --- | --- | --- | --- | --- | --- | --- |
|  | |  | Univariable | | |  | Multivariable | |  | Univariable | | |  | Multivariable | |
| **Covariate** | |  | **C-index** | **HR (95% CI)** | **P value** |  | **HR (95% CI)** | **P value** |  | **C-index** | **HR (95% CI)** | **P value** |  | **HR (95% CI)** | **P value** |
| Sex (Male) | |  | 0.542 | 0.72 (0.49-1.05) | 0.086 |  | 0.67 (0.45-0.99) | 0.045 |  | 0.59 | 2.08 (1.17-3.7) | 0.013 |  | 1.42 (0.79-2.57) | 0.244 |
| Baseline age (years) | |  | 0.543 | 0.99 (0.97-1) | 0.146 |  | 0.99 (0.98-1.01) | 0.423 |  | 0.716 | 1.07 (1.04-1.1) | <0.001 |  | 1.09 (1.06-1.12) | <0.001 |
| BMI (kg/m^2^) | |  | 0.519 | 1.02 (0.98-1.06) | 0.419 |  | 1.02 (0.98-1.06) | 0.29 |  | 0.555 | 1.04 (0.98-1.09) | 0.2 |  | 1.01 (0.94-1.08) | 0.823 |
| Smoking (Yes) | |  | 0.592 | 2.42 (1.66-3.54) | <0.001 |  | 2.15 (1.44-3.21) | <0.001 |  | 0.735 | 7.92 (4.33-14.51) | <0.001 |  | 10.54 (5.38-20.65) | <0.001 |
| Income | |  | 0.522 | 0.95 (0.87-1.04) | 0.304 |  | 0.96 (0.87-1.05) | 0.375 |  | 0.733 | 0.63 (0.53-0.76) | <0.001 |  | 0.73 (0.6-0.87) | <0.001 |
| Education level | |  | 0.554 |  |  |  |  |  |  | 0.621 |  |  |  |  |  |
|  | Low |  |  | 1 | - |  | 1 | - |  |  | 1 | - |  | 1 | - |
|  | Middle |  |  | 1.34 (0.86-2.08) | 0.19 |  | 1.49 (0.96-2.33) | 0.078 |  |  | 0.66 (0.36-1.23) | 0.193 |  | 0.58 (0.31-1.1) | 0.097 |
|  | High |  |  | 0.82 (0.5-1.34) | 0.431 |  | 1.06 (0.64-1.77) | 0.823 |  |  | 0.3 (0.14-0.67) | 0.003 |  | 0.63 (0.28-1.43) | 0.267 |
| Gut microbiome | |  | 0.643 | 1.5 (1.29-1.75) | <0.001 |  | 1.4 (1.19-1.64) | <0.001 |  | 0.814 | 1.38 (1.28-1.48) | <0.001 |  | 1.16 (1.05-1.27) | 0.002 |
