# Supplementary figures and images for: The gut microbiome is a significant risk factor for future chronic lung disease
Source: J Allergy Clin Immunol. 2023 Apr;151(4):943–52. doi: 10.1016/j.jaci.2022.12.810 (PMC10109092; doi:10.1016/j.jaci.2022.12.810)

(A)

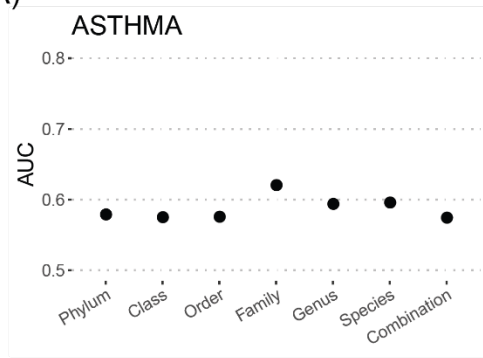

(B)

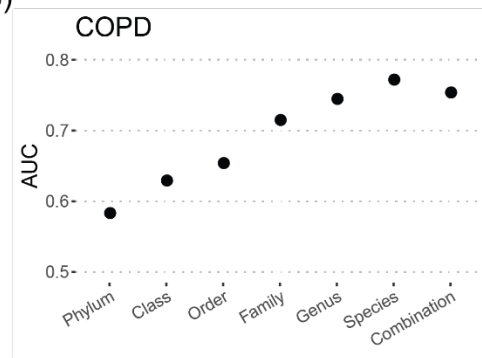

(C)

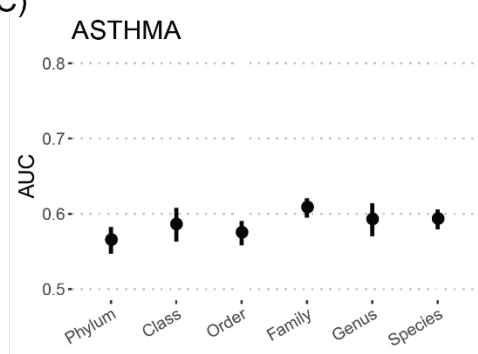

(D)

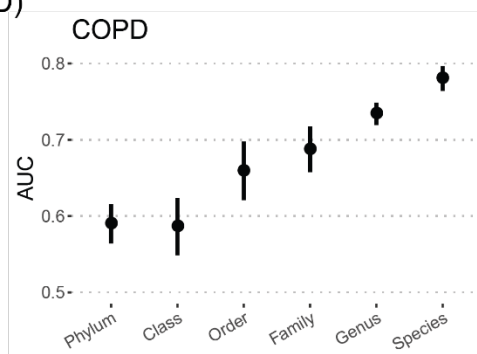

Supplement: Supplementary Fig S1 [file mmc5.pdf]
